# Supplementary figures and images for: Feeding ecology of broadbill swordfish (Xiphias gladius) in the California current
Source: PLoS One. 2023 Feb 16;18(2):e0258011. doi: 10.1371/journal.pone.0258011 (PMC9934375; doi:10.1371/journal.pone.0258011)

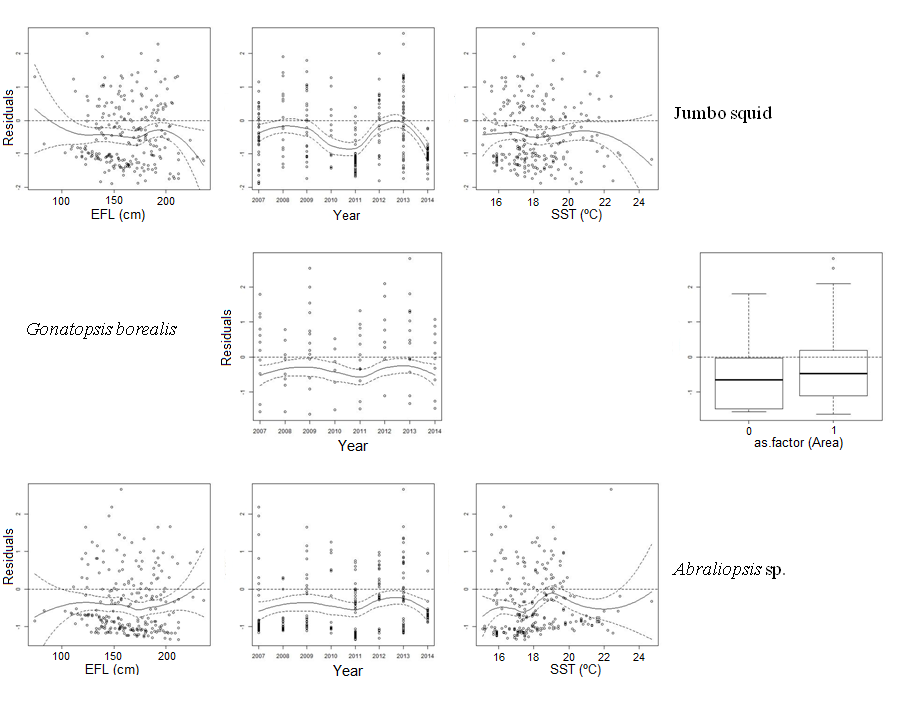

Supplement: S1 Fig — (TIF) [file pone.0258011.s001.tif]

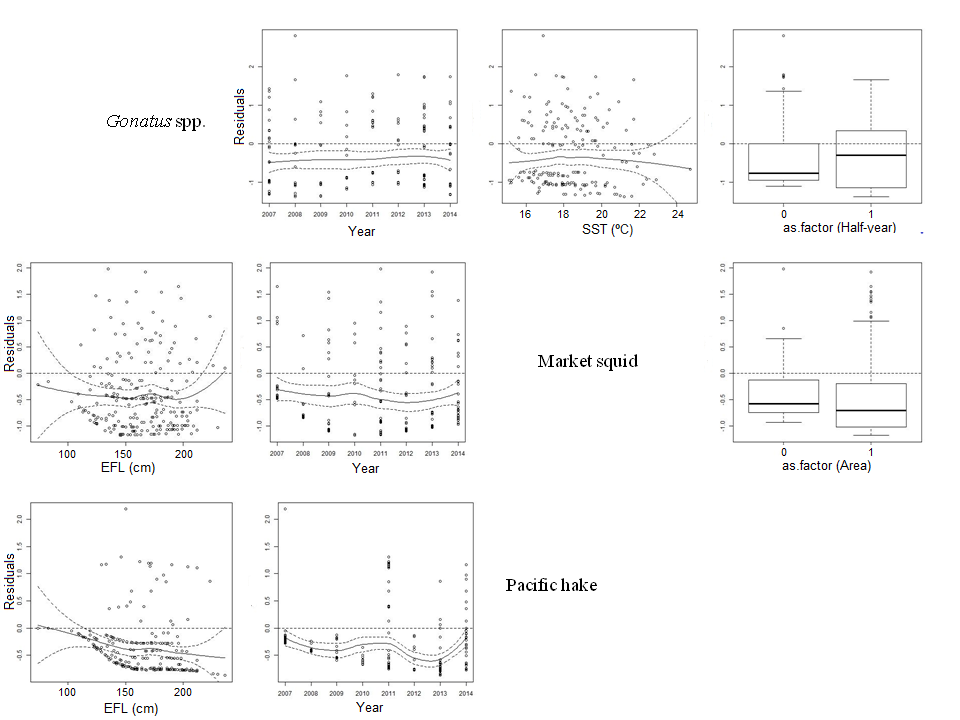

Supplement: S2 Fig — (TIF) [file pone.0258011.s002.tif]

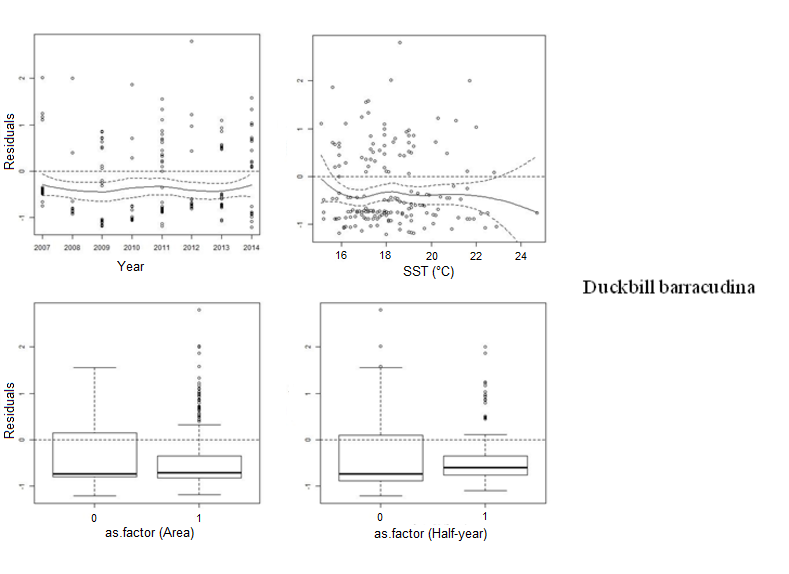

Supplement: S3 Fig — (TIF) [file pone.0258011.s003.tif]
