# Supplementary material for: Feeding ecology of broadbill swordfish (Xiphias gladius) in the California current
Source: PLoS One. 2023 Feb 16;18(2):e0258011. doi: 10.1371/journal.pone.0258011 (PMC9934375; doi:10.1371/journal.pone.0258011)
Supplement: S1 Table — A total of 148 stomachs containing food was examined. Prey items are shown by decreasing GII value. See methods for description of the measured values. (DOCX) [file pone.0258011.s004.docx]

**Table S1.** Quantitative prey composition of the broadbill swordfish (EFL < 165 cm) in the California Current. A total of 148 stomachs containing food was examined. Prey items are shown by decreasing GII value. See methods for description of the measured values.

| **Prey Species** | ***W* (g)** | ***%W*** | ***N*** | ***%N*** | ***F*** | ***%F*** | **GII** | **%GII** | **IRI** | **%IRI** | **%PSIRI** |
| --- | --- | --- | --- | --- | --- | --- | --- | --- | --- | --- | --- |
| **Jumbo squid, *Dosidicus gigas*** | 24962.3 | 42.17 | 369 | 15.19 | 71 | 47.97 | 60.82 | 35.11 | 2751.88 | 41.32 | 28.68 |
| **Boreopacific gonate squid, *Gonatopsis borealis*** | 11328.4 | 19.14 | 370 | 15.23 | 89 | 60.14 | 54.56 | 31.5 | 2066.91 | 31.03 | 17.19 |
| ***Abraliopsis* sp.** | 15.3 | 0.03 | 218 | 8.97 | 53 | 35.81 | 25.87 | 14.94 | 322.32 | 4.84 | 4.50 |
| **Duckbill barracudina, *Magnisudis atlantica*** | 2350 | 3.97 | 122 | 5.02 | 49 | 33.11 | 24.31 | 14.03 | 297.73 | 4.47 | 4.50 |
| ***Gonatus* spp.** | 116.7 | 0.2 | 117 | 4.82 | 47 | 31.76 | 21.23 | 12.26 | 159.23 | 2.39 | 2.51 |
| **Market squid, *Doryteuthis opalescens*** | 483.4 | 0.82 | 209 | 8.6 | 39 | 26.35 | 20.65 | 11.92 | 248.26 | 3.73 | 4.71 |
| **Chubby pearleye, *Rosenblattichthys volucris*** | 664 | 1.12 | 134 | 5.52 | 33 | 22.3 | 16.71 | 9.65 | 148.02 | 2.22 | 3.32 |
| **Unidentified Teleostei** | 306.3 | 0.52 | 69 | 2.84 | 35 | 23.65 | 15.59 | 9 | 79.42 | 1.19 | 1.68 |
| **Pacific hake, *Merluccius productus*** | 7994.1 | 13.51 | 57 | 2.35 | 16 | 10.81 | 15.39 | 8.89 | 171.37 | 2.57 | 7.93 |
| ***Nansenia* spp.** | 490 | 0.83 | 111 | 4.57 | 28 | 18.92 | 14.04 | 8.11 | 102.12 | 1.53 | 2.70 |
| **Slender barracudina, *Lestidiops ringens*** | 265.6 | 0.45 | 68 | 2.8 | 19 | 12.84 | 9.29 | 5.36 | 41.7 | 0.63 | 1.63 |
| **Pacific saury, *Cololabis saira*** | 1332.6 | 2.25 | 69 | 2.84 | 14 | 9.46 | 8.4 | 4.85 | 48.17 | 0.72 | 2.55 |
| **Sunbeam lampfish, *Lampadena urophaos*** | 201.9 | 0.34 | 42 | 1.73 | 18 | 12.16 | 8.22 | 4.74 | 25.18 | 0.38 | 1.04 |
| **Unidentified Scopelarchidae** | 420.3 | 0.71 | 74 | 3.05 | 15 | 10.14 | 8.02 | 4.63 | 38.07 | 0.57 | 1.88 |
| **Jack mackerel, *Trachurus symmetricus*** | 1771.3 | 2.99 | 22 | 0.91 | 13 | 8.78 | 7.32 | 4.23 | 34.24 | 0.51 | 1.95 |
| ***Onychoteuthis borealijaponica*** | 28 | 0.05 | 18 | 0.74 | 16 | 10.81 | 6.7 | 3.87 | 8.52 | 0.13 | 0.40 |
| **Pacific sardine, *Sardinops sagax*** | 1033.6 | 1.75 | 42 | 1.73 | 12 | 8.11 | 6.69 | 3.86 | 28.18 | 0.42 | 1.74 |
| **Unidentified Eucarida** | 5.5 | 0.01 | 154 | 6.34 | 6 | 4.05 | 6.01 | 3.47 | 25.74 | 0.39 | 3.18 |
| **Cock-eyed squid, *Histioteuthis heteropsis*** | 794.7 | 1.34 | 25 | 1.03 | 9 | 6.08 | 4.88 | 2.82 | 14.42 | 0.22 | 1.19 |
| **Flowervase jewell squid, *Histioteuthis dofleini*** | 554.6 | 0.94 | 18 | 0.74 | 10 | 6.76 | 4.87 | 2.81 | 11.34 | 0.17 | 0.84 |
| **Pacific mackerel, *Scomber japonicus*** | 589.4 | 1 | 29 | 1.19 | 9 | 6.08 | 4.78 | 2.76 | 13.32 | 0.2 | 1.10 |
| **Pacific pomfret, *Brama japonica*** | 578.1 | 0.98 | 11 | 0.45 | 8 | 5.41 | 3.95 | 2.28 | 7.73 | 0.12 | 0.72 |
| **Unidentified Teuthoidea** | 189.9 | 0.32 | 7 | 0.29 | 7 | 4.73 | 3.08 | 1.78 | 2.88 | 0.04 | 0.31 |
| **Striped mullet, *Mugil cephalus*** | 1726.4 | 2.92 | 7 | 0.29 | 3 | 2.03 | 3.02 | 1.74 | 6.5 | 0.1 | 1.61 |
| **Spotted barracudina, *Arctozenus risso*** | 29.8 | 0.05 | 6 | 0.25 | 5 | 3.38 | 2.12 | 1.23 | 1 | 0.02 | 0.15 |
| **Sharpchin barracudina, *Stemonosudis macrura*** | 8.8 | 0.01 | 8 | 0.33 | 4 | 2.7 | 1.76 | 1.02 | 0.93 | 0.01 | 0.17 |
| **Bigfin lampfish, *Symbolophorus californiensis*** | 5.4 | 0.01 | 6 | 0.25 | 4 | 2.7 | 1.71 | 0.99 | 0.69 | 0.01 | 0.13 |
| ***Argonauta* sp.** | 7.5 | 0.01 | 4 | 0.16 | 4 | 2.7 | 1.66 | 0.96 | 0.48 | 0.01 | 0.09 |
| **Mexican lampfish, *Triphoturus mexicanus*** | <0.1 | <0.01 | 4 | 0.16 | 4 | 2.7 | 1.66 | 0.96 | 0.45 | 0.01 | 0.09 |
| ***Histioteuthis* spp.** | 5.2 | 0.01 | 4 | 0.16 | 3 | 2.03 | 1.27 | 0.73 | 0.35 | 0.01 | 0.09 |
| ***Cranchia scabra*** | 4.5 | 0.01 | 4 | 0.16 | 3 | 2.03 | 1.27 | 0.73 | 0.35 | 0.01 | 0.09 |
| **Northern anchovy, *Engraulis mordax*** | 1.6 | <0.01 | 4 | 0.16 | 3 | 2.03 | 1.27 | 0.73 | 0.34 | 0.01 | 0.09 |
| **King-of-the-salmon, *Trachipterus altivelis*** | 341.7 | 0.58 | 6 | 0.25 | 2 | 1.35 | 1.26 | 0.73 | 1.11 | 0.02 | 0.42 |
| **Smalleye squaretail, *Tetragonurus cuvieri*** | 161.9 | 0.27 | 3 | 0.12 | 2 | 1.35 | 1.01 | 0.58 | 0.54 | 0.01 | 0.20 |
| **Paralepididae, Barracudinas** | 43.4 | 0.07 | 3 | 0.12 | 2 | 1.35 | 0.89 | 0.52 | 0.27 | <0.01 | 0.10 |
| **Unidentified Tunicata** | 1.5 | <0.01 | 2 | 0.08 | 2 | 1.35 | 0.83 | 0.48 | 0.11 | <0.01 | 0.05 |
| ***Octopoteuthis* sp.** | <0.1 | <0.01 | 2 | 0.08 | 2 | 1.35 | 0.83 | 0.48 | 0.11 | <0.01 | 0.05 |
| **Albacore, *Thunnus alalunga*** | 371.6 | 0.63 | 1 | 0.04 | 1 | 0.68 | 0.78 | 0.45 | 0.45 | 0.01 | 0.34 |
| **Shortbelly rockfish, *Sebastes jordani*** | 0.4 | <0.01 | 2 | 0.08 | 1 | 0.68 | 0.44 | 0.25 | 0.06 | <0.01 | 0.05 |
| **California smoothtongue, *Leuroglossus stilbius*** | <0.1 | <0.01 | 2 | 0.08 | 1 | 0.68 | 0.44 | 0.25 | 0.06 | <0.01 | 0.05 |
| **Robust clubhook squid, *Onykia robusta*** | 6 | 0.01 | 1 | 0.04 | 1 | 0.68 | 0.42 | 0.24 | 0.03 | <0.01 | 0.03 |
| **Northern lampfish, *Stenobrachius leucopsarus*** | <0.1 | <0.01 | 1 | 0.04 | 1 | 0.68 | 0.41 | 0.24 | 0.03 | <0.01 | 0.03 |
| ***Onychoteuthis* sp.** | <0.1 | <0.01 | 1 | 0.04 | 1 | 0.68 | 0.41 | 0.24 | 0.03 | <0.01 | 0.03 |
| ***Mastigoteuthis dentata*** | <0.1 | <0.01 | 1 | 0.04 | 1 | 0.68 | 0.41 | 0.24 | 0.03 | <0.01 | 0.03 |
| ***Octopus* spp.** | <0.1 | <0.01 | 1 | 0.04 | 1 | 0.68 | 0.41 | 0.24 | 0.03 | <0.01 | 0.03 |
| **California flashlightfish, *Protomyctophum crockeri*** | <0.1 | <0.01 | 1 | 0.04 | 1 | 0.68 | 0.41 | 0.24 | 0.03 | <0.01 | 0.03 |
