# Supplementary material for: Feeding ecology of broadbill swordfish (Xiphias gladius) in the California current
Source: PLoS One. 2023 Feb 16;18(2):e0258011. doi: 10.1371/journal.pone.0258011 (PMC9934375; doi:10.1371/journal.pone.0258011)
Supplement: S2 Table — A total of 140 stomachs containing food was examined. Prey items are shown by decreasing GII value. See methods for description of the measured values. (DOCX) [file pone.0258011.s005.docx]

**Table S2.** Quantitative prey composition of the broadbill swordfish (EFL ≥ 165 cm) in the California Current. A total of 140 stomachs containing food was examined. Prey items are shown by decreasing GII value. See methods for description of the measured values.

| **Prey Species** | ***W* (g)** | ***%W*** | ***N*** | ***%N*** | ***F*** | ***%F*** | **GII** | **%GII** | **IRI** | **%IRI** | **%PSIRI** |
| --- | --- | --- | --- | --- | --- | --- | --- | --- | --- | --- | --- |
| **Jumbo squid, *Dosidicus gigas*** | 106404 | 57.27 | 685 | 25.3 | 101 | 72.14 | 89.33 | 51.57 | 5957.28 | 62.8 | 41.29 |
| **Boreopacific gonate squid, *Gonatopsis borealis*** | 8502.1 | 4.58 | 506 | 18.69 | 90 | 64.29 | 50.55 | 29.18 | 1495.83 | 15.77 | 11.64 |
| ***Abraliopsis* sp.** | 29.7 | 0.02 | 237 | 8.76 | 63 | 45 | 31.04 | 17.92 | 394.7 | 4.16 | 4.39 |
| ***Gonatus* spp.** | 64.9 | 0.03 | 181 | 6.69 | 62 | 44.29 | 29.45 | 17 | 297.66 | 3.14 | 3.36 |
| **Pacific hake, *Merluccius productus*** | 27143.9 | 14.61 | 271 | 10.01 | 32 | 22.86 | 27.41 | 15.83 | 562.77 | 5.93 | 12.31 |
| **Market squid, *Doryteuthis opalescens*** | 559.6 | 0.3 | 270 | 9.97 | 47 | 33.57 | 25.31 | 14.62 | 344.96 | 3.64 | 5.14 |
| **Duckbill barracudina, *Magnisudis atlantica*** | 2218.6 | 1.19 | 96 | 3.55 | 35 | 25 | 17.17 | 9.91 | 118.51 | 1.25 | 2.37 |
| **Unidentified Teleostei** | 2010.6 | 1.08 | 50 | 1.85 | 29 | 20.71 | 13.65 | 7.88 | 60.68 | 0.64 | 1.47 |
| **Luvar, *Luvarus imperialis*** | 19258.5 | 10.37 | 18 | 0.66 | 7 | 5 | 9.26 | 5.34 | 55.15 | 0.58 | 5.52 |
| **Jack mackerel, *Trachurus symmetricus*** | 4896.9 | 2.64 | 50 | 1.85 | 15 | 10.71 | 8.77 | 5.07 | 48.03 | 0.51 | 2.25 |
| ***Onychoteuthis borealijaponica*** | 628.2 | 0.34 | 39 | 1.44 | 18 | 12.86 | 8.45 | 4.88 | 22.87 | 0.24 | 0.89 |
| **Pacific pomfret, *Brama japonica*** | 4366.1 | 2.35 | 29 | 1.07 | 15 | 10.71 | 8.16 | 4.71 | 36.66 | 0.39 | 1.71 |
| **Pacific sardine, *Sardinops sagax*** | 789.5 | 0.42 | 35 | 1.29 | 14 | 10 | 6.77 | 3.91 | 17.18 | 0.18 | 0.86 |
| **Chubby pearleye, *Rosenblattichthys volucris*** | 137.8 | 0.07 | 25 | 0.92 | 15 | 10.71 | 6.76 | 3.9 | 10.69 | 0.11 | 0.50 |
| **King-of-the-salmon, *Trachipterus altivelis*** | 5235.7 | 2.82 | 19 | 0.7 | 11 | 7.86 | 6.57 | 3.79 | 27.66 | 0.29 | 1.76 |
| **Cock-eyed squid, *Histioteuthis heteropsis*** | 517.5 | 0.28 | 27 | 1 | 9 | 6.43 | 4.45 | 2.57 | 8.2 | 0.09 | 0.64 |
| **Slender barracudina, *Lestidiops ringens*** | 50.1 | 0.03 | 23 | 0.85 | 9 | 6.43 | 4.22 | 2.44 | 5.64 | 0.06 | 0.44 |
| **Pacific mackerel, *Scomber japonicus*** | 1591.3 | 0.86 | 37 | 1.37 | 7 | 5 | 4.17 | 2.41 | 11.12 | 0.12 | 1.12 |
| **Pacific saury, *Cololabis saira*** | 34.2 | 0.02 | 7 | 0.26 | 6 | 4.29 | 2.63 | 1.52 | 1.19 | 0.01 | 0.14 |
| **Unidentified Scopelarchidae** | 56.6 | 0.03 | 12 | 0.44 | 5 | 3.57 | 2.34 | 1.35 | 1.69 | 0.02 | 0.24 |
| **Unidentified Teuthoidea** | 12.1 | 0.01 | 8 | 0.3 | 5 | 3.57 | 2.24 | 1.29 | 1.08 | 0.01 | 0.16 |
| **Flowervase jewell squid, *Histioteuthis dofleini*** | 5.5 | <0.01 | 7 | 0.26 | 5 | 3.57 | 2.21 | 1.28 | 0.93 | 0.01 | 0.14 |
| ***Histioteuthis* spp.** | 51.5 | 0.03 | 5 | 0.18 | 5 | 3.57 | 2.18 | 1.26 | 0.76 | 0.01 | 0.10 |
| ***Nansenia* spp.** | 20.9 | 0.01 | 13 | 0.48 | 4 | 2.86 | 1.93 | 1.12 | 1.4 | 0.01 | 0.25 |
| ***Argonauta* sp.** | 5.6 | <0.01 | 4 | 0.15 | 4 | 2.86 | 1.74 | 1 | 0.43 | <0.01 | 0.08 |
| **Shortbelly rockfish, *Sebastes jordani*** | 2.1 | <0.01 | 4 | 0.15 | 4 | 2.86 | 1.74 | 1 | 0.43 | <0.01 | 0.08 |
| **Unidentified Euphausiidae** | 3 | <0.01 | 6 | 0.22 | 3 | 2.14 | 1.37 | 0.79 | 0.48 | 0.01 | 0.12 |
| **Spotted barracudina, *Arctozenus risso*** | 36.9 | 0.02 | 7 | 0.26 | 2 | 1.43 | 0.99 | 0.57 | 0.4 | <0.01 | 0.14 |
| **Paralepididae, Barracudinas** | 67.9 | 0.04 | 4 | 0.15 | 2 | 1.43 | 0.93 | 0.54 | 0.26 | <0.01 | 0.10 |
| ***Japetella* sp.** | <0.1 | <0.01 | 4 | 0.15 | 2 | 1.43 | 0.91 | 0.53 | 0.21 | <0.01 | 0.08 |
| **Robust clubhook squid, *Onykia robusta*** | 37.3 | 0.02 | 3 | 0.11 | 2 | 1.43 | 0.9 | 0.52 | 0.19 | <0.01 | 0.07 |
| ***Octopus rubescens*** | <0.1 | <0.01 | 2 | 0.07 | 2 | 1.43 | 0.87 | 0.5 | 0.11 | <0.01 | 0.04 |
| ***Chiroteuthis calyx*** | <0.1 | <0.01 | 2 | 0.07 | 2 | 1.43 | 0.87 | 0.5 | 0.11 | <0.01 | 0.04 |
| **California smoothtongue, *Leuroglossus stilbius*** | <0.1 | <0.01 | 2 | 0.07 | 2 | 1.43 | 0.87 | 0.5 | 0.11 | <0.01 | 0.04 |
| **Splitnose rockfish, *Sebastes diploproa*** | 924.2 | 0.5 | 2 | 0.07 | 1 | 0.71 | 0.74 | 0.43 | 0.41 | <0.01 | 0.29 |
| ***Onychoteuthis* sp.** | <0.1 | <0.01 | 3 | 0.11 | 1 | 0.71 | 0.48 | 0.28 | 0.08 | <0.01 | 0.06 |
| **Halfmoon, *Medialuna californiensis*** | 81 | 0.04 | 1 | 0.04 | 1 | 0.71 | 0.46 | 0.26 | 0.06 | <0.01 | 0.04 |
| **Dogtooth lampfish, *Ceratoscopelus townsendi*** | 1.5 | <0.01 | 2 | 0.07 | 1 | 0.71 | 0.46 | 0.26 | 0.05 | <0.01 | 0.04 |
| ***Leachia dislocate*** | <0.1 | <0.01 | 2 | 0.07 | 1 | 0.71 | 0.46 | 0.26 | 0.05 | <0.01 | 0.04 |
| **Pacific bonito, *Sarda chiliensis*** | 25.8 | 0.01 | 1 | 0.04 | 1 | 0.71 | 0.44 | 0.26 | 0.04 | <0.01 | 0.03 |
| **Striped mullet, *Mugil cephalus*** | 11.4 | 0.01 | 1 | 0.04 | 1 | 0.71 | 0.44 | 0.25 | 0.03 | <0.01 | 0.03 |
| ***Auxis* sp.** | 4.7 | <0.01 | 1 | 0.04 | 1 | 0.71 | 0.44 | 0.25 | 0.03 | <0.01 | 0.03 |
| **Unidentified Tunicata** | 2 | <0.01 | 1 | 0.04 | 1 | 0.71 | 0.43 | 0.25 | 0.03 | <0.01 | 0.03 |
| **Northern lampfish, *Stenobrachius leucopsarus*** | <0.1 | <0.01 | 1 | 0.04 | 1 | 0.71 | 0.43 | 0.25 | 0.03 | <0.01 | 0.03 |
| ***Cranchia scabra*** | <0.1 | <0.01 | 1 | 0.04 | 1 | 0.71 | 0.43 | 0.25 | 0.03 | <0.01 | 0.03 |
| **Bigfin lampfish, *Symbolophorus californiensis*** | <0.1 | <0.01 | 1 | 0.04 | 1 | 0.71 | 0.43 | 0.25 | 0.03 | <0.01 | 0.03 |
| **California headlightfish, *Diaphus theta*** | <0.1 | <0.01 | 1 | 0.04 | 1 | 0.71 | 0.43 | 0.25 | 0.03 | <0.01 | 0.03 |
| **Unidentified Isopoda** | <0.1 | <0.01 | 1 | 0.04 | 1 | 0.71 | 0.43 | 0.25 | 0.03 | <0.01 | 0.03 |
