# Supplementary material for: Feeding ecology of broadbill swordfish (Xiphias gladius) in the California current
Source: PLoS One. 2023 Feb 16;18(2):e0258011. doi: 10.1371/journal.pone.0258011 (PMC9934375; doi:10.1371/journal.pone.0258011)
Supplement: S3 Table — Values of mean GII, bootstrapped 95% CIs and % bootstrap runs in which each prey type was in the smaller of two size categories of swordfish. If more than 95% (or fewer than 5%) of runs show the prey type was more important in the smaller size category of swordfish than in the larger category, the difference is considered to be significant. S = small (EFL < 165 cm), M = medium (EFL ≥ 165 cm). These results are generally consistent with inferences from non-overlap of 95% CIs. (DOCX) [file pone.0258011.s006.docx]

**Table S3.** Comparison of GII for the main prey species between small and medium broadbill swordfish. Values of mean GII, bootstrapped 95% CIs and % bootstrap runs in which each prey type was in the smaller of two size categories of swordfish. If more than 95% (or fewer than 5%) of runs show the prey type was more important in the smaller size category of swordfish than in the larger category, we consider the difference to be significant. S = small (EFL < 165 cm), M = medium (EFL ≥ 165 cm). These results are generally consistent with inferences from non-overlap of 95% CIs.

|  | **Mean GII and (95% CI)** | | **Bootstrap results** |
| --- | --- | --- | --- |
| **Prey taxa** | **S** | **M** | **% runs S>M** |
| **Jumbo squid** | 26.03  (20.95 - 32.07) | 89.24  (79.60 -100.75) | 0.0 |
| ***Gonatopsis borealis*** | 55.15  (47.76 - 64.13) | 50.39  (43.46 - 57.65) | 79.4 |
| ***Abraliopsis* sp.** | 26.03  (20.95 - 32.07) | 31.05  (26.09 - 36.16) | 13.1 |
| ***Gonatus* spp.** | 21.33  (16.38 - 24.88) | 29.28  (23.54 - 35.11) | 1.9 |
| **Market squid** | 20.35  (15.82 - 26.35) | 25.56  (19.17 - 31.14) | 12.3 |
| **Pacific hake** | 15.53  (7.26 - 23.39) | 27.49  (18.91 - 38.27) | 4.4 |
