# Supplementary material for: Feeding ecology of broadbill swordfish (Xiphias gladius) in the California current
Source: PLoS One. 2023 Feb 16;18(2):e0258011. doi: 10.1371/journal.pone.0258011 (PMC9934375; doi:10.1371/journal.pone.0258011)
Supplement: S4 Table — A total of 199 stomachs containing food was examined. Prey items are shown by decreasing GII value. See methods for description of the measured values. (DOCX) [file pone.0258011.s007.docx]

**Table S4.** Quantitative prey composition of the broadbill swordfish within the SCB subregion. A total of 199 stomachs containing food was examined. Prey items are shown by decreasing GII value. See methods for description of the measured values.

| **Prey Species** | ***W* (g)** | ***%W*** | ***N*** | ***%N*** | ***F*** | ***%F*** | **GII** | **%GII** | **IRI** | **%IRI** | **%PSIRI** |
| --- | --- | --- | --- | --- | --- | --- | --- | --- | --- | --- | --- |
| **Jumbo squid, *Dosidicus gigas*** | 103327.5 | 66.57 | 839 | 22.34 | 130 | 65.33 | 89.04 | 51.41 | 5807.71 | 66.65 | 44.46 |
| **Boreopacific gonate squid, *Gonatopsis borealis*** | 8805.9 | 5.67 | 508 | 13.53 | 119 | 59.8 | 45.61 | 26.33 | 1148.02 | 13.17 | 9.60 |
| ***Abraliopsis* sp.** | 44.3 | 0.03 | 308 | 8.2 | 88 | 44.22 | 30.28 | 17.48 | 363.88 | 4.18 | 4.12 |
| **Market squid, *Doryteuthis opalescens*** | 1130.1 | 0.73 | 487 | 12.97 | 68 | 34.17 | 27.63 | 15.95 | 467.93 | 5.37 | 6.85 |
| ***Gonatus* spp.** | 138 | 0.09 | 237 | 6.31 | 81 | 40.7 | 27.19 | 15.7 | 260.45 | 2.99 | 3.20 |
| **Chubby pearleye, *Rosenblattichthys volucris*** | 791.2 | 0.51 | 162 | 4.31 | 48 | 24.12 | 16.71 | 9.65 | 116.33 | 1.33 | 2.41 |
| **Duckbill barracudina, *Magnisudis atlantica*** | 2330.4 | 1.5 | 122 | 3.25 | 48 | 24.12 | 16.67 | 9.62 | 114.56 | 1.31 | 2.38 |
| **Pacific hake, *Merluccius productus*** | 15720.2 | 10.13 | 150 | 3.99 | 22 | 11.06 | 14.54 | 8.39 | 156.11 | 1.79 | 7.06 |
| **Unidentified Teleostei** | 603.8 | 0.39 | 82 | 2.18 | 46 | 23.12 | 14.83 | 8.56 | 59.46 | 0.68 | 1.29 |
| **Jack mackerel, *Trachurus symmetricus*** | 2189.8 | 1.41 | 54 | 1.44 | 21 | 10.55 | 7.74 | 4.47 | 30.06 | 0.34 | 1.43 |
| **Pacific sardine, *Sardinops sagax*** | 1340.4 | 0.86 | 62 | 1.65 | 21 | 10.55 | 7.54 | 4.36 | 26.53 | 0.3 | 1.26 |
| **Unidentified Scopelarchidae** | 476.9 | 0.31 | 86 | 2.29 | 20 | 10.05 | 7.3 | 4.22 | 26.1 | 0.3 | 1.30 |
| ***Onychoteuthis borealijaponica*** | 157.7 | 0.1 | 36 | 0.96 | 21 | 10.55 | 6.7 | 3.87 | 11.19 | 0.13 | 0.53 |
| ***Nansenia* spp.** | 193.2 | 0.12 | 87 | 2.32 | 18 | 9.05 | 6.63 | 3.83 | 22.08 | 0.25 | 1.22 |
| **Slender barracudina, *Lestidiops ringens*** | 242.8 | 0.16 | 75 | 2 | 18 | 9.05 | 6.47 | 3.73 | 19.48 | 0.22 | 1.08 |
| **Pacific saury, *Cololabis saira*** | 1244.9 | 0.8 | 69 | 1.84 | 15 | 7.54 | 5.88 | 3.39 | 19.89 | 0.23 | 1.32 |
| **Pacific mackerel, *Scomber japonicus*** | 1969.5 | 1.27 | 64 | 1.7 | 14 | 7.04 | 5.78 | 3.34 | 20.91 | 0.24 | 1.49 |
| **King-of-the-salmon, *Trachipterus altivelis*** | 5057.4 | 3.26 | 23 | 0.61 | 12 | 6.03 | 5.72 | 3.3 | 23.34 | 0.27 | 1.94 |
| **Unidentified Eucarida** | 4.5 | <0.01 | 129 | 3.43 | 5 | 2.51 | 3.44 | 1.98 | 8.64 | 0.1 | 1.72 |
| **Cock-eyed squid, *Histioteuthis heteropsis*** | 924.4 | 0.6 | 29 | 0.77 | 8 | 4.02 | 3.11 | 1.8 | 5.5 | 0.06 | 0.69 |
| **Flowervase jewell squid, *Histioteuthis dofleini*** | 120.9 | 0.08 | 12 | 0.32 | 9 | 4.52 | 2.84 | 1.64 | 1.8 | 0.02 | 0.20 |
| **Luvar, *Luvarus imperialis*** | 4789.4 | 3.09 | 4 | 0.11 | 3 | 1.51 | 2.71 | 1.57 | 4.81 | 0.06 | 1.60 |
| **Unidentified Teuthoidea** | 186.4 | 0.12 | 9 | 0.24 | 7 | 3.52 | 2.24 | 1.29 | 1.27 | 0.01 | 0.18 |
| ***Argonauta* sp.** | 13 | 0.01 | 7 | 0.19 | 7 | 3.52 | 2.14 | 1.24 | 0.69 | 0.01 | 0.10 |
| **Sunbeam lampfish, *Lampadena urophaos*** | 48.5 | 0.03 | 12 | 0.32 | 6 | 3.02 | 1.94 | 1.12 | 1.06 | 0.01 | 0.18 |
| **Striped mullet, *Mugil cephalus*** | 1737.8 | 1.12 | 8 | 0.21 | 4 | 2.01 | 1.93 | 1.11 | 2.68 | 0.03 | 0.67 |
| ***Histioteuthis* spp.** | 56.6 | 0.04 | 7 | 0.19 | 6 | 3.02 | 1.87 | 1.08 | 0.67 | 0.01 | 0.12 |
| **Spotted barracudina, *Arctozenus risso*** | 29.8 | 0.02 | 7 | 0.19 | 6 | 3.02 | 1.86 | 1.07 | 0.62 | 0.01 | 0.11 |
| ***Octopoteuthis* sp.** | 2.1 | <0.01 | 5 | 0.13 | 5 | 2.51 | 1.53 | 0.88 | 0.34 | <0.01 | 0.07 |
| **Pacific pomfret, *Brama japonica*** | 327.4 | 0.21 | 6 | 0.16 | 4 | 2.01 | 1.37 | 0.79 | 0.75 | 0.01 | 0.19 |
| **Bigfin lampfish, *Symbolophorus californiensis*** | 5.4 | <0.01 | 6 | 0.16 | 4 | 2.01 | 1.25 | 0.72 | 0.33 | <0.01 | 0.09 |
| ***Cranchia scabra*** | 4.5 | <0.01 | 5 | 0.13 | 4 | 2.01 | 1.24 | 0.72 | 0.27 | <0.01 | 0.07 |
| **Barracudinas, Paralepididae** | 111.3 | 0.07 | 7 | 0.19 | 3 | 1.51 | 1.02 | 0.59 | 0.39 | <0.01 | 0.13 |
| **Northern anchovy, *Engraulis mordax*** | 1.6 | <0.01 | 4 | 0.11 | 3 | 1.51 | 0.93 | 0.54 | 0.16 | <0.01 | 0.06 |
| **Mexican lampfish, *Triphoturus mexicanus*** | <0.1 | <0.01 | 3 | 0.08 | 3 | 1.51 | 0.92 | 0.53 | 0.12 | <0.01 | 0.05 |
| **Splitnose rockfish, *Sebastes diploproa*** | 924.2 | 0.6 | 2 | 0.05 | 1 | 0.5 | 0.66 | 0.38 | 0.33 | <0.01 | 0.33 |
| ***Japetella* sp.** | <0.1 | <0.01 | 4 | 0.11 | 2 | 1.01 | 0.64 | 0.37 | 0.11 | <0.01 | 0.06 |
| **Robust clubhook squid*, Onykia robusta*** | 42.3 | 0.03 | 2 | 0.05 | 2 | 1.01 | 0.63 | 0.36 | 0.08 | <0.01 | 0.04 |
| **California smoothtongue, *Leuroglossus stilbius*** | <0.1 | <0.01 | 3 | 0.08 | 2 | 1.01 | 0.63 | 0.36 | 0.08 | <0.01 | 0.05 |
| ***Octopus rubescens*** | <0.1 | <0.01 | 2 | 0.05 | 2 | 1.01 | 0.61 | 0.35 | 0.05 | <0.01 | 0.03 |
| ***Chiroteuthis calyx*** | <0.1 | <0.01 | 2 | 0.05 | 2 | 1.01 | 0.61 | 0.35 | 0.05 | <0.01 | 0.03 |
| ***Sebastes* spp.** | 3 | <0.01 | 8 | 0.21 | 1 | 0.5 | 0.41 | 0.24 | 0.11 | <0.01 | 0.11 |
| **Sharpchin barracudina, *Stemonosudis macrura*** | 1.6 | <0.01 | 4 | 0.11 | 1 | 0.5 | 0.35 | 0.2 | 0.05 | <0.01 | 0.06 |
| **Halfmoon, *Medialuna californiensis*** | 81 | 0.05 | 1 | 0.03 | 1 | 0.5 | 0.34 | 0.19 | 0.04 | <0.01 | 0.04 |
| **Smalleye squaretail, *Tetragonurus cuvieri*** | 13.7 | 0.01 | 2 | 0.05 | 1 | 0.5 | 0.33 | 0.19 | 0.03 | <0.01 | 0.03 |
| **Dogtooth lampfish, *Ceratoscopelus townsendi*** | 1.5 | <0.01 | 2 | 0.05 | 1 | 0.5 | 0.32 | 0.19 | 0.03 | <0.01 | 0.03 |
| **Shortbelly rockfish, *Sebastes jordani*** | 0.4 | <0.01 | 2 | 0.05 | 1 | 0.5 | 0.32 | 0.19 | 0.03 | <0.01 | 0.03 |
| ***Leachia dislocata*** | <0.1 | <0.01 | 2 | 0.05 | 1 | 0.5 | 0.32 | 0.19 | 0.03 | <0.01 | 0.03 |
| **Pacific bonito, *Sarda chiliensis*** | 25.8 | 0.02 | 1 | 0.03 | 1 | 0.5 | 0.32 | 0.18 | 0.02 | <0.01 | 0.03 |
| ***Auxis* sp.** | 4.7 | <0.01 | 1 | 0.03 | 1 | 0.5 | 0.31 | 0.18 | 0.01 | <0.01 | 0.02 |
| **Unidentified Euphausiidae** | 1.5 | <0.01 | 1 | 0.03 | 1 | 0.5 | 0.31 | 0.18 | 0.01 | <0.01 | 0.02 |
| **Unidentified Tunicata** | 0.3 | <0.01 | 1 | 0.03 | 1 | 0.5 | 0.31 | 0.18 | 0.01 | <0.01 | 0.02 |
| **Northern lampfish, *Stenobrachius leucopsarus*** | <0.1 | <0.01 | 1 | 0.03 | 1 | 0.5 | 0.31 | 0.18 | 0.01 | <0.01 | 0.02 |
| ***Onychoteuthis* sp.** | <0.1 | <0.01 | 1 | 0.03 | 1 | 0.5 | 0.31 | 0.18 | 0.01 | <0.01 | 0.02 |
| ***Mastigoteuthis dentata*** | <0.1 | <0.01 | 1 | 0.03 | 1 | 0.5 | 0.31 | 0.18 | 0.01 | <0.01 | 0.02 |
| ***Octopus* spp.** | <0.1 | <0.01 | 1 | 0.03 | 1 | 0.5 | 0.31 | 0.18 | 0.01 | <0.01 | 0.02 |
| **California flashlightfish, *Protomyctophum crockeri*** | <0.1 | <0.01 | 1 | 0.03 | 1 | 0.5 | 0.31 | 0.18 | 0.01 | <0.01 | 0.02 |
