# Supplementary material for: Feeding ecology of broadbill swordfish (Xiphias gladius) in the California current
Source: PLoS One. 2023 Feb 16;18(2):e0258011. doi: 10.1371/journal.pone.0258011 (PMC9934375; doi:10.1371/journal.pone.0258011)
Supplement: S5 Table — A total of 93 stomachs containing food was examined. Prey items are shown by decreasing GII value. See methods for description of the measured values. (DOCX) [file pone.0258011.s008.docx]

**Table S5.** Quantitative prey composition of the broadbill swordfish beyond the SCB subregion. A total of 93 stomachs containing food was examined. Prey items are shown by decreasing GII value. See methods for description of the measured values.

| **Prey Species** | ***W* (g)** | ***%W*** | ***N*** | ***%N*** | ***F*** | ***%F*** | **GII** | **%GII** | **IRI** | **%IRI** | **%PSIRI** |
| --- | --- | --- | --- | --- | --- | --- | --- | --- | --- | --- | --- |
| **Boreopacific gonate squid, *Gonatopsis borealis*** | 11143.9 | 12.07 | 376 | 25.27 | 63 | 67.74 | 60.67 | 35.03 | 2529.19 | 35.91 | 18.67 |
| **Jumbo squid, *Dosidicus gigas*** | 28565.2 | 30.93 | 222 | 14.92 | 43 | 46.24 | 53.17 | 30.7 | 2119.97 | 30.1 | 22.93 |
| **Pacific hake, *Merluccius productus*** | 20639.9 | 22.35 | 181 | 12.16 | 27 | 29.03 | 36.69 | 21.18 | 1002 | 14.23 | 17.26 |
| **Duckbill barracudina, *Magnisudis atlantica*** | 2238.1 | 2.42 | 96 | 6.45 | 36 | 38.71 | 27.47 | 15.86 | 343.55 | 4.88 | 4.44 |
| ***Abraliopsis* sp.** | 0.8 | <0.01 | 156 | 10.48 | 29 | 31.18 | 24.06 | 13.89 | 326.94 | 4.64 | 5.25 |
| ***Gonatus* spp.** | 43.6 | 0.05 | 62 | 4.17 | 29 | 31.18 | 20.44 | 11.8 | 131.4 | 1.87 | 2.11 |
| **Pacific pomfret, *Brama japonica*** | 4914.2 | 5.32 | 35 | 2.35 | 20 | 21.51 | 16.85 | 9.73 | 165.02 | 2.34 | 3.84 |
| **Market squid, *Doryteuthis opalescens*** | 317.6 | 0.34 | 51 | 3.43 | 20 | 21.51 | 14.59 | 8.43 | 81.1 | 1.15 | 1.89 |
| **Unidentified Teleostei** | 1713.1 | 1.85 | 37 | 2.49 | 20 | 21.51 | 14.92 | 8.62 | 93.37 | 1.32 | 2.17 |
| **Luvar, *Luvarus imperialis*** | 14469.1 | 15.67 | 14 | 0.94 | 4 | 4.3 | 12.07 | 6.97 | 71.43 | 1.01 | 8.31 |
| ***Nansenia* spp.** | 317.7 | 0.34 | 37 | 2.49 | 14 | 15.05 | 10.33 | 5.96 | 42.61 | 0.6 | 1.42 |
| ***Onychoteuthis borealijaponica*** | 498.8 | 0.54 | 24 | 1.61 | 14 | 15.05 | 9.93 | 5.74 | 32.41 | 0.46 | 1.08 |
| **Sunbeam lampfish, *Lampadena urophaos*** | 153.4 | 0.17 | 30 | 2.02 | 12 | 12.9 | 8.71 | 5.03 | 28.16 | 0.4 | 1.10 |
| **Jack mackerel, *Trachurus symmetricus*** | 4478.4 | 4.85 | 18 | 1.21 | 7 | 7.53 | 7.84 | 4.53 | 45.61 | 0.65 | 3.03 |
| **Slender barracudina, *Lestidiops ringens*** | 87.2 | 0.09 | 17 | 1.14 | 11 | 11.83 | 7.54 | 4.35 | 14.63 | 0.21 | 0.62 |
| **Cock-eyed squid, *Histioteuthis heteropsis*** | 387.8 | 0.42 | 23 | 1.55 | 10 | 10.75 | 7.34 | 4.24 | 21.14 | 0.3 | 0.99 |
| **Flowervase jewell squid, *Histioteuthis dofleini*** | 439.1 | 0.48 | 13 | 0.87 | 6 | 6.45 | 4.5 | 2.6 | 8.7 | 0.12 | 0.68 |
| **Pacific saury, *Cololabis saira*** | 121.9 | 0.13 | 7 | 0.47 | 6 | 6.45 | 4.07 | 2.35 | 3.89 | 0.06 | 0.30 |
| **Pacific sardine, *Sardinops sagax*** | 482.7 | 0.52 | 15 | 1.01 | 5 | 5.38 | 3.99 | 2.3 | 8.23 | 0.12 | 0.77 |
| **Unidentified Teuthoidea** | 15.6 | 0.02 | 6 | 0.4 | 5 | 5.38 | 3.35 | 1.93 | 2.26 | 0.03 | 0.21 |
| **Sharpchin barracudina, *Stemonosudis macrura*** | 7.2 | 0.01 | 4 | 0.27 | 3 | 3.23 | 2.02 | 1.17 | 0.89 | 0.01 | 0.14 |
| **Unidentified Eucarida** | 1 | <0.01 | 25 | 1.68 | 1 | 1.08 | 1.59 | 0.92 | 1.81 | 0.03 | 0.85 |
| **Spotted barracudina, *Arctozenus risso*** | 38.1 | 0.04 | 7 | 0.47 | 2 | 2.15 | 1.54 | 0.89 | 1.1 | 0.02 | 0.26 |
| **Pacific mackerel, *Scomber japonicus*** | 211.2 | 0.23 | 2 | 0.13 | 2 | 2.15 | 1.45 | 0.84 | 0.78 | 0.01 | 0.18 |
| **Euphausiidae** | 1.5 | <0.01 | 5 | 0.34 | 2 | 2.15 | 1.44 | 0.83 | 0.73 | 0.01 | 0.18 |
| **Unidentified Tunicata** | 3.2 | <0.01 | 2 | 0.13 | 2 | 2.15 | 1.32 | 0.76 | 0.3 | <0.01 | 0.07 |
| ***Histioteuthis* spp.** | 0.1 | <0.01 | 2 | 0.13 | 2 | 2.15 | 1.32 | 0.76 | 0.29 | <0.01 | 0.07 |
| **King-of-the-salmon, *Trachipterus altivelis*** | 520 | 0.56 | 2 | 0.13 | 1 | 1.08 | 1.02 | 0.59 | 0.75 | 0.01 | 0.35 |
| **Albacore, *Thunnus alalunga*** | 371.6 | 0.4 | 1 | 0.07 | 1 | 1.08 | 0.89 | 0.51 | 0.5 | 0.01 | 0.24 |
| **Chubby pearleye, *Rosenblattichthys volucris*** | 19.4 | 0.02 | 4 | 0.27 | 1 | 1.08 | 0.79 | 0.46 | 0.31 | <0.01 | 0.15 |
| **Smalleye squaretail, *Tetragonurus cuvieri*** | 148.2 | 0.16 | 1 | 0.07 | 1 | 1.08 | 0.75 | 0.43 | 0.24 | <0.01 | 0.12 |
| ***Onychoteuthis* sp.** | <0.1 | <0.01 | 3 | 0.2 | 1 | 1.08 | 0.74 | 0.43 | 0.22 | <0.01 | 0.11 |
| **Robust clubhook squid*, Onykia robusta*** | 1 | <0.01 | 2 | 0.13 | 1 | 1.08 | 0.7 | 0.4 | 0.15 | <0.01 | 0.07 |
| ***Argonauta* sp.** | 0.1 | <0.01 | 1 | 0.07 | 1 | 1.08 | 0.66 | 0.38 | 0.07 | <0.01 | 0.04 |
| **Northern lampfish, *Stenobrachius leucopsarus*** | <0.1 | <0.01 | 1 | 0.07 | 1 | 1.08 | 0.66 | 0.38 | 0.07 | <0.01 | 0.04 |
| ***Octopoteuthis* sp.** | <0.1 | <0.01 | 1 | 0.07 | 1 | 1.08 | 0.66 | 0.38 | 0.07 | <0.01 | 0.04 |
| **Bigfin lampfish, *Symbolophorus californiensis*** | <0.1 | <0.01 | 1 | 0.07 | 1 | 1.08 | 0.66 | 0.38 | 0.07 | <0.01 | 0.04 |
| **Mexican lampfish, *Triphoturus mexicanus*** | <0.1 | <0.01 | 1 | 0.07 | 1 | 1.08 | 0.66 | 0.38 | 0.07 | <0.01 | 0.04 |
| **California headlightfish, *Diaphus theta*** | <0.1 | <0.01 | 1 | 0.07 | 1 | 1.08 | 0.66 | 0.38 | 0.07 | <0.01 | 0.04 |
| **California smoothtongue, *Leuroglossus stilbius*** | <0.1 | <0.01 | 1 | 0.07 | 1 | 1.08 | 0.66 | 0.38 | 0.07 | <0.01 | 0.04 |
| **Unidentified Isopoda** | <0.1 | <0.01 | 1 | 0.07 | 1 | 1.08 | 0.66 | 0.38 | 0.07 | <0.01 | 0.04 |
