# Supplementary material for: Feeding ecology of broadbill swordfish (Xiphias gladius) in the California current
Source: PLoS One. 2023 Feb 16;18(2):e0258011. doi: 10.1371/journal.pone.0258011 (PMC9934375; doi:10.1371/journal.pone.0258011)
Supplement: S6 Table — Values of mean GII, bootstrapped 95% CIs and % bootstrap runs in which each prey type was in each of two categories of swordfish. If more than 95% (or fewer than 5%) of runs show the prey type was more important in one region than the other, the difference is considered to be significant. East = within the SCB subregion, West = beyond the SCB subregion. These results are generally consistent with inferences from non-overlap of 95% CIs. (DOCX) [file pone.0258011.s009.docx]

**Table S6.** Comparison of GII for the main prey species between broadbill swordfish within and beyond the SCB region. Values of mean GII, bootstrapped 95% CIs and % bootstrap runs in which each prey type was in each of two categories of swordfish. If more than 95% (or fewer than 5%) of runs show the prey type was more important in one region than the other, we consider the difference to be significant. East = within the SCB subregion, West = beyond the SCB subregion. These results are generally consistent with inferences from non-overlap of 95% CIs.

|  | **Mean GII and (95% CI)** | | **Bootstrap results** |
| --- | --- | --- | --- |
| **Prey taxa** | **East** | **West** | **% runs East>West** |
| **Jumbo squid** | 88.86 (78.79 - 96.77) | 53.03 (40.69 - 65.54) | 100.0 |
| ***Gonatopsis borealis*** | 45.73 (40.68 - 51.53) | 60.82 (50.98 - 70.92) | 0.3 |
| ***Abraliopsis* sp.** | 30.16 (25.21 - 35.31) | 23.95 (16.84 - 30.95) | 93.1 |
| ***Gonatus* spp.** | 27.37 (22.83 - 32.63) | 20.33 (14.53 - 25.91) | 96.3 |
| **Market squid** | 27.54 (23.30 - 33.70) | 14.88 (9.42 - 21.13) | 99.8 |
| **Pacific hake** | 14.50 (7.54 - 22.62) | 36.67 (23.90 - 50.35) | 0.0 |
