# Supplementary material for: Feeding ecology of broadbill swordfish (Xiphias gladius) in the California current
Source: PLoS One. 2023 Feb 16;18(2):e0258011. doi: 10.1371/journal.pone.0258011 (PMC9934375; doi:10.1371/journal.pone.0258011)
Supplement: S7 Table — A total of 47 stomachs containing food was examined. Prey items are shown by decreasing GII value. See methods for description of the measured values. (DOCX) [file pone.0258011.s010.docx]

**Table S7.** Quantitative prey composition of the broadbill swordfish during year 2007 in the California Current. A total of 47 stomachs containing food was examined. Prey items are shown by decreasing GII value. See methods for description of the measured values.

| **Prey Species** | ***W* (g)** | ***%W*** | ***N*** | ***%N*** | ***F*** | ***%F*** | **GII** | **%GII** | **IRI** | **%IRI** | **%PSIRI** |
| --- | --- | --- | --- | --- | --- | --- | --- | --- | --- | --- | --- |
| **Jumbo squid, *Dosidicus gigas*** | 20152.9 | 76.22 | 276 | 38.82 | 40 | 85.11 | 115.55 | 66.71 | 9790.47 | 77.6 | 57.52 |
| **Boreopacific gonate squid, *Gonatopsis borealis*** | 3091.7 | 11.69 | 157 | 22.08 | 27 | 57.45 | 52.67 | 30.41 | 1940.24 | 15.38 | 16.89 |
| ***Abraliopsis* sp.** | 0.1 | <0.01 | 96 | 13.5 | 13 | 27.66 | 23.76 | 13.72 | 373.48 | 2.96 | 6.76 |
| ***Gonatus* spp.** | 1.6 | 0.01 | 47 | 6.61 | 15 | 31.91 | 22.25 | 12.84 | 211.17 | 1.67 | 3.31 |
| **Unidentified Teleostei** | 56.6 | 0.21 | 15 | 2.11 | 11 | 23.4 | 14.85 | 8.58 | 54.39 | 0.43 | 1.16 |
| **Chubby pearleye, *Rosenblattichthys volucris*** | 87 | 0.33 | 17 | 2.39 | 9 | 19.15 | 12.63 | 7.29 | 52.09 | 0.41 | 1.36 |
| **Duckbill barracudina, *Magnisudis atlantica*** | 307.2 | 1.16 | 20 | 2.81 | 7 | 14.89 | 10.89 | 6.29 | 59.2 | 0.47 | 1.99 |
| **Striped mullet, *Mugil cephalus*** | 1726.4 | 6.53 | 7 | 0.98 | 3 | 6.38 | 8.02 | 4.63 | 47.96 | 0.38 | 3.76 |
| **Jack mackerel, *Trachurus symmetricus*** | 483.4 | 1.83 | 4 | 0.56 | 4 | 8.51 | 6.29 | 3.63 | 20.35 | 0.16 | 1.20 |
| **Pacific saury, *Cololabis saira*** | 78 | 0.3 | 7 | 0.98 | 4 | 8.51 | 5.65 | 3.26 | 10.89 | 0.09 | 0.64 |
| **Sharpchin barracudina*, Stemonosudis macrura*** | 8.8 | 0.03 | 8 | 1.13 | 4 | 8.51 | 5.58 | 3.22 | 9.86 | 0.08 | 0.58 |
| **Unidentified Scopelarchidae** | 268.8 | 1.02 | 14 | 1.97 | 3 | 6.38 | 5.41 | 3.12 | 19.06 | 0.15 | 1.50 |
| **Market squid, *Doryteuthis opalescens*** | 19.4 | 0.07 | 5 | 0.7 | 4 | 8.51 | 5.36 | 3.1 | 6.61 | 0.05 | 0.39 |
| **Unidentified Teuthoidea** | 8.2 | 0.03 | 5 | 0.7 | 4 | 8.51 | 5.34 | 3.08 | 6.25 | 0.05 | 0.37 |
| **Paralepididae, Barracudinas** | 72.6 | 0.27 | 5 | 0.7 | 2 | 4.26 | 3.02 | 1.74 | 4.16 | 0.03 | 0.49 |
| **Northern anchovy, *Engraulis mordax*** | 0.7 | <0.01 | 3 | 0.42 | 2 | 4.26 | 2.7 | 1.56 | 1.81 | 0.01 | 0.22 |
| **Sunbeam lampfish, *Lampadena urophaos*** | 6.8 | 0.03 | 2 | 0.28 | 2 | 4.26 | 2.63 | 1.52 | 1.31 | 0.01 | 0.16 |
| **Northern lampfish, *Stenobrachius leucopsarus*** | <0.1 | <0.01 | 2 | 0.28 | 2 | 4.26 | 2.62 | 1.51 | 1.2 | 0.01 | 0.15 |
| **Cock-eyed squid, *Histioteuthis heteropsis*** | <0.1 | <0.01 | 11 | 1.55 | 1 | 2.13 | 2.12 | 1.22 | 3.29 | 0.03 | 0.78 |
| **Pacific hake, *Merluccius productus*** | 39 | 0.15 | 2 | 0.28 | 1 | 2.13 | 1.48 | 0.85 | 0.91 | 0.01 | 0.22 |
| **Slender barracudina, *Lestidiops ringens*** | <0.1 | <0.01 | 2 | 0.28 | 1 | 2.13 | 1.39 | 0.8 | 0.6 | <0.01 | 0.15 |
| **Pacific bonito, *Sarda chiliensis*** | 25.8 | 0.1 | 1 | 0.14 | 1 | 2.13 | 1.37 | 0.79 | 0.51 | <0.01 | 0.12 |
| **Bigfin lampfish, *Symbolophorus californiensis*** | 3.7 | 0.01 | 1 | 0.14 | 1 | 2.13 | 1.32 | 0.76 | 0.33 | <0.01 | 0.08 |
| **Pacific pomfret, *Brama japonica*** | 1.7 | 0.01 | 1 | 0.14 | 1 | 2.13 | 1.31 | 0.76 | 0.31 | <0.01 | 0.08 |
| **Flowervase jewell squid, *Histioteuthis dofleini*** | <0.1 | <0.01 | 1 | 0.14 | 1 | 2.13 | 1.31 | 0.76 | 0.3 | <0.01 | 0.08 |
| ***Onychoteuthis* sp.** | <0.1 | <0.01 | 1 | 0.14 | 1 | 2.13 | 1.31 | 0.76 | 0.3 | <0.01 | 0.08 |
| ***Mastigoteuthis dentata*** | <0.1 | <0.01 | 1 | 0.14 | 1 | 2.13 | 1.31 | 0.76 | 0.3 | <0.01 | 0.08 |
