# Supplementary material for: Feeding ecology of broadbill swordfish (Xiphias gladius) in the California current
Source: PLoS One. 2023 Feb 16;18(2):e0258011. doi: 10.1371/journal.pone.0258011 (PMC9934375; doi:10.1371/journal.pone.0258011)
Supplement: S8 Table — A total of 16 stomachs containing food was examined. Prey items are shown by decreasing GII value. See methods for description of the measured values. (DOCX) [file pone.0258011.s011.docx]

**Table S8.** Quantitative prey composition of the broadbill swordfish during year 2008 in the California Current. A total of 16 stomachs containing food was examined. Prey items are shown by decreasing GII value. See methods for description of the measured values.

| **Prey Species** | ***W* (g)** | ***%W*** | ***N*** | ***%N*** | ***F*** | ***%F*** | **GII** | **%GII** | **IRI** | **%IRI** | **%PSIRI** |
| --- | --- | --- | --- | --- | --- | --- | --- | --- | --- | --- | --- |
| **Jumbo squid, *Dosidicus gigas*** | 30536.6 | 95.75 | 110 | 44.35 | 14 | 87.5 | 131.41 | 75.87 | 12258.77 | 82.3 | 70.05 |
| **Boreopacific gonate squid, *Gonatopsis borealis*** | 1022.5 | 3.21 | 45 | 18.15 | 11 | 68.75 | 52.02 | 30.03 | 1467.9 | 9.85 | 10.68 |
| ***Gonatus* spp.** | 3.5 | 0.01 | 47 | 18.95 | 8 | 50 | 39.82 | 22.99 | 948.14 | 6.36 | 9.48 |
| ***Abraliopsis* sp.** | <0.1 | <0.01 | 14 | 5.65 | 3 | 18.75 | 14.08 | 8.13 | 105.85 | 0.71 | 2.83 |
| **Duckbill barracudina, *Magnisudis atlantica*** | 213.5 | 0.67 | 8 | 3.23 | 2 | 12.5 | 9.47 | 5.47 | 48.69 | 0.33 | 1.95 |
| **Market squid, *Doryteuthis opalescens*** | <0.1 | <0.01 | 4 | 1.61 | 2 | 12.5 | 8.15 | 4.7 | 20.16 | 0.14 | 0.81 |
| **Pacific sardine, *Sardinops sagax*** | 23.5 | 0.07 | 4 | 1.61 | 1 | 6.25 | 4.58 | 2.65 | 10.54 | 0.07 | 0.84 |
| **Chubby pearleye, *Rosenblattichthys volucris*** | 13.9 | 0.04 | 3 | 1.21 | 1 | 6.25 | 4.33 | 2.5 | 7.83 | 0.05 | 0.63 |
| **Barracudinas, Paralepididae** | 38.7 | 0.12 | 2 | 0.81 | 1 | 6.25 | 4.14 | 2.39 | 5.8 | 0.04 | 0.47 |
| **Unidentified Scopelarchidae** | 9.1 | 0.03 | 2 | 0.81 | 1 | 6.25 | 4.09 | 2.36 | 5.22 | 0.04 | 0.42 |
| **Pacific saury, *Cololabis saira*** | 5.8 | 0.02 | 2 | 0.81 | 1 | 6.25 | 4.08 | 2.36 | 5.15 | 0.03 | 0.42 |
| **Pacific mackerel, *Scomber japonicus*** | 3.1 | 0.01 | 2 | 0.81 | 1 | 6.25 | 4.08 | 2.36 | 5.1 | 0.03 | 0.41 |
| **Jack mackerel, *Trachurus symmetricus*** | 14.6 | 0.05 | 1 | 0.4 | 1 | 6.25 | 3.87 | 2.23 | 2.81 | 0.02 | 0.23 |
| ***Auxis* sp.** | 4.7 | 0.01 | 1 | 0.4 | 1 | 6.25 | 3.85 | 2.22 | 2.61 | 0.02 | 0.21 |
| **Unidentified Teleostei** | 3 | 0.01 | 1 | 0.4 | 1 | 6.25 | 3.85 | 2.22 | 2.58 | 0.02 | 0.21 |
| **Northern anchovy, *Engraulis mordax*** | 0.9 | <0.01 | 1 | 0.4 | 1 | 6.25 | 3.84 | 2.22 | 2.54 | 0.02 | 0.21 |
| **Flowervase jewell squid, *Histioteuthis dofleini*** | <0.1 | <0.01 | 1 | 0.4 | 1 | 6.25 | 3.84 | 2.22 | 2.52 | 0.02 | 0.21 |
