# Supplementary material for: Feeding ecology of broadbill swordfish (Xiphias gladius) in the California current
Source: PLoS One. 2023 Feb 16;18(2):e0258011. doi: 10.1371/journal.pone.0258011 (PMC9934375; doi:10.1371/journal.pone.0258011)
Supplement: S9 Table — A total of 37 stomachs containing food was examined. Prey items are shown by decreasing GII value. See methods for description of the measured values. (DOCX) [file pone.0258011.s012.docx]

**Table S9.** Quantitative prey composition of the broadbill swordfish during year 2009 in the California Current. A total of 37 stomachs containing food was examined. Prey items are shown by decreasing GII value. See methods for description of the measured values.

| **Prey Species** | ***W* (g)** | ***%W*** | ***N*** | ***%N*** | ***F*** | ***%F*** | **GII** | **%GII** | **IRI** | **%IRI** | **%PSIRI** |
| --- | --- | --- | --- | --- | --- | --- | --- | --- | --- | --- | --- |
| **Boreopacific gonate squid, *Gonatopsis borealis*** | 6784.2 | 20.42 | 378 | 43.85 | 33 | 89.19 | 88.6 | 51.15 | 5732.56 | 42.92 | 32.14 |
| **Jumbo squid, *Dosidicus gigas*** | 21434.7 | 64.53 | 132 | 15.31 | 26 | 70.27 | 86.67 | 50.04 | 5610.27 | 42 | 39.92 |
| ***Abraliopsis* sp.** | 0.4 | <0.01 | 91 | 10.56 | 21 | 56.76 | 38.86 | 22.44 | 599.24 | 4.49 | 5.29 |
| ***Gonatus* spp.** | 26.1 | 0.08 | 51 | 5.92 | 17 | 45.95 | 29.99 | 17.31 | 275.45 | 2.06 | 3.00 |
| **Market squid, *Doryteuthis opalescens*** | 489.1 | 1.47 | 94 | 10.9 | 14 | 37.84 | 28.99 | 16.74 | 468.32 | 3.51 | 6.19 |
| **Duckbill barracudina, *Magnisudis atlantica*** | 1260.1 | 3.79 | 45 | 5.22 | 15 | 40.54 | 28.61 | 16.52 | 365.42 | 2.74 | 4.51 |
| **Pacific pomfret, *Brama japonica*** | 2832.1 | 8.53 | 16 | 1.86 | 8 | 21.62 | 18.48 | 10.67 | 224.47 | 1.68 | 5.20 |
| **Unidentified Scopelarchidae** | 76.6 | 0.23 | 20 | 2.32 | 8 | 21.62 | 13.96 | 8.06 | 55.15 | 0.41 | 1.28 |
| **Unidentified Teleostei** | 19.7 | 0.06 | 8 | 0.93 | 4 | 10.81 | 6.81 | 3.93 | 10.67 | 0.08 | 0.50 |
| ***Nansenia* spp.** | 54.7 | 0.16 | 7 | 0.81 | 2 | 5.41 | 3.68 | 2.13 | 5.28 | 0.04 | 0.49 |
| **Pacific sardine, *Sardinops sagax*** | 46.6 | 0.14 | 2 | 0.23 | 2 | 5.41 | 3.34 | 1.93 | 2.01 | 0.02 | 0.19 |
| **Jack mackerel, *Trachurus symmetricus*** | 22.7 | 0.07 | 2 | 0.23 | 2 | 5.41 | 3.29 | 1.9 | 1.62 | 0.01 | 0.15 |
| **Unidentified Teuthoidea** | <0.1 | <0.01 | 2 | 0.23 | 2 | 5.41 | 3.25 | 1.88 | 1.25 | 0.01 | 0.12 |
| **Smalleye squaretail, *Tetragonurus cuvieri*** | 148.2 | 0.45 | 1 | 0.12 | 1 | 2.7 | 1.88 | 1.09 | 1.52 | 0.01 | 0.29 |
| ***Onychoteuthis* sp.** | <0.1 | <0.01 | 3 | 0.35 | 1 | 2.7 | 1.76 | 1.02 | 0.94 | 0.01 | 0.18 |
| **Flowervase jewell squid, *Histioteuthis dofleini*** | 2.8 | 0.01 | 2 | 0.23 | 1 | 2.7 | 1.7 | 0.98 | 0.65 | <0.01 | 0.12 |
| **Shortbelly rockfish, *Sebastes jordani*** | 0.4 | <0.01 | 2 | 0.23 | 1 | 2.7 | 1.7 | 0.98 | 0.63 | <0.01 | 0.12 |
| **Striped mullet, *Mugil cephalus*** | 11.4 | 0.03 | 1 | 0.12 | 1 | 2.7 | 1.65 | 0.95 | 0.41 | <0.01 | 0.08 |
| **Chubby pearleye, *Rosenblattichthys volucris*** | 8.9 | 0.03 | 1 | 0.12 | 1 | 2.7 | 1.64 | 0.95 | 0.39 | <0.01 | 0.08 |
| **Pacific mackerel, *Scomber japonicus*** | 0.3 | <0.01 | 1 | 0.12 | 1 | 2.7 | 1.63 | 0.94 | 0.32 | <0.01 | 0.07 |
| ***Octopoteuthis* sp.** | <0.1 | <0.01 | 1 | 0.12 | 1 | 2.7 | 1.63 | 0.94 | 0.31 | <0.01 | 0.07 |
| **East Pacific Red Octopus*, Octopus rubescens*** | <0.1 | <0.01 | 1 | 0.12 | 1 | 2.7 | 1.63 | 0.94 | 0.31 | <0.01 | 0.07 |
| ***Japetella* sp.** | <0.1 | <0.01 | 1 | 0.12 | 1 | 2.7 | 1.63 | 0.94 | 0.31 | <0.01 | 0.07 |
