# Supplementary material for: Feeding ecology of broadbill swordfish (Xiphias gladius) in the California current
Source: PLoS One. 2023 Feb 16;18(2):e0258011. doi: 10.1371/journal.pone.0258011 (PMC9934375; doi:10.1371/journal.pone.0258011)
Supplement: S10 Table — A total of 12 stomachs containing food was examined. Prey items are shown by decreasing GII value. See methods for description of the measured values. (DOCX) [file pone.0258011.s013.docx]

**Table S10.** Quantitative prey composition of the broadbill swordfish during year 2010 in the California Current. A total of 12 stomachs containing food was examined. Prey items are shown by decreasing GII value. See methods for description of the measured values.

| **Prey Species** | ***W* (g)** | ***%W*** | ***N*** | ***%N*** | ***F*** | ***%F*** | **GII** | **%GII** | **IRI** | **%IRI** | **%PSIRI** |
| --- | --- | --- | --- | --- | --- | --- | --- | --- | --- | --- | --- |
| **Jumbo squid, *Dosidicus gigas*** | 5392.1 | 72 | 12 | 5.5 | 7 | 58.3 | 78.4 | 45.29 | 4522.33 | 44.86 | 38.76 |
| **Boreopacific gonate squid, *Gonatopsis borealis*** | 476.5 | 6.36 | 31 | 14.2 | 10 | 83.3 | 60 | 34.64 | 1715.39 | 17.02 | 10.29 |
| **Market squid, *Doryteuthis opalescens*** | 34.1 | 0.46 | 69 | 31.7 | 8 | 66.7 | 57 | 32.92 | 2140.48 | 21.23 | 16.06 |
| ***Abraliopsis* sp.** | 0.1 | <0.01 | 28 | 12.8 | 6 | 50 | 36.3 | 20.95 | 642.24 | 6.37 | 6.43 |
| ***Nansenia* spp.** | 4 | 0.05 | 25 | 11.5 | 3 | 25 | 21.1 | 12.17 | 288.04 | 2.86 | 5.76 |
| **Pacific sardine, *Sardinops sagax*** | 572.9 | 7.65 | 24 | 11 | 2 | 16.7 | 20.4 | 11.78 | 311.02 | 3.09 | 9.33 |
| **Duckbill barracudina, *Magnisudis atlantica*** | 208.6 | 2.79 | 5 | 2.29 | 3 | 25 | 17.4 | 10.03 | 127 | 1.26 | 2.54 |
| **Jack mackerel, *Trachurus symmetricus*** | 369.8 | 4.94 | 3 | 1.38 | 2 | 16.7 | 13.3 | 7.66 | 105.26 | 1.04 | 3.16 |
| **Pacific pomfret, *Brama japonica*** | 314.4 | 4.2 | 3 | 1.38 | 2 | 16.7 | 12.8 | 7.41 | 92.93 | 0.92 | 2.79 |
| **Pacific mackerel, *Scomber japonicus*** | 95.6 | 1.28 | 5 | 2.29 | 2 | 16.7 | 11.7 | 6.75 | 59.51 | 0.59 | 1.79 |
| **Chubby pearleye, *Rosenblattichthys volucris*** | 10.3 | 0.14 | 6 | 2.75 | 2 | 16.7 | 11.3 | 6.52 | 48.16 | 0.48 | 1.45 |
| ***Histioteuthis* spp.** | 0.2 | <0.01 | 2 | 0.92 | 1 | 8.33 | 5.34 | 3.08 | 7.67 | 0.08 | 0.47 |
| **Pacific saury, *Cololabis saira*** | 7.7 | 0.1 | 1 | 0.46 | 1 | 8.33 | 5.14 | 2.96 | 4.68 | 0.05 | 0.28 |
| **Sunbeam lampfish, *Lampadena urophaos*** | 0.5 | 0.01 | 1 | 0.46 | 1 | 8.33 | 5.08 | 2.93 | 3.88 | 0.04 | 0.24 |
| **Unidentified Scopelarchidae** | <0.1 | <0.01 | 1 | 0.46 | 1 | 8.33 | 5.08 | 2.93 | 3.82 | 0.04 | 0.24 |
| **Unidentified Teleostei** | <0.1 | <0.01 | 1 | 0.46 | 1 | 8.33 | 5.08 | 2.93 | 3.82 | 0.04 | 0.24 |
| **California flashlightfish, *Protomyctophum crockeri*** | <0.1 | <0.01 | 1 | 0.46 | 1 | 8.33 | 5.08 | 2.93 | 3.82 | 0.04 | 0.24 |
