# Supplementary material for: Feeding ecology of broadbill swordfish (Xiphias gladius) in the California current
Source: PLoS One. 2023 Feb 16;18(2):e0258011. doi: 10.1371/journal.pone.0258011 (PMC9934375; doi:10.1371/journal.pone.0258011)
Supplement: S11 Table — A total of 54 stomachs containing food was examined. Prey items are shown by decreasing GII value. See methods for description of the measured values. (DOCX) [file pone.0258011.s014.docx]

**Table S11.** Quantitative prey composition of the broadbill swordfish during year 2011 in the California Current. A total of 54 stomachs containing food was examined. Prey items are shown by decreasing GII value. See methods for description of the measured values.

|  |  |  |  |  |  |  |  |  |  |  |
| --- | --- | --- | --- | --- | --- | --- | --- | --- | --- | --- |

| **Prey Species** | ***W* (g)** | ***%W*** | ***N*** | ***%N*** | ***F*** | ***%F*** | **GII** | **%GII** | **IRI** | **%IRI** | **%PSIRI** |
| --- | --- | --- | --- | --- | --- | --- | --- | --- | --- | --- | --- |
| **Pacific hake, *Merluccius productus*** | 22111.3 | 59.76 | 172 | 17.32 | 21 | 38.89 | 66.95 | 38.66 | 2997.54 | 45.85 | 38.54 |
| **Boreopacific gonate squid, *Gonatopsis borealis*** | 2792 | 7.55 | 72 | 7.25 | 30 | 55.56 | 40.62 | 23.45 | 822.03 | 12.57 | 7.40 |
| **Market squid, *Doryteuthis opalescens*** | 381.1 | 1.03 | 138 | 13.9 | 25 | 46.3 | 35.35 | 20.41 | 691.08 | 10.57 | 7.47 |
| **Duckbill barracudina, *Magnisudis atlantica*** | 918.9 | 2.48 | 62 | 6.24 | 24 | 44.44 | 30.7 | 17.72 | 387.88 | 5.93 | 4.36 |
| ***Abraliopsis* sp.** | 1.4 | <0.01 | 55 | 5.54 | 23 | 42.59 | 27.79 | 16.05 | 236.08 | 3.61 | 2.78 |
| **Unidentified Teleostei** | 1954.3 | 5.28 | 45 | 4.53 | 15 | 27.78 | 21.7 | 12.53 | 272.59 | 4.1 | 4.91 |
| ***Nansenia* spp.** | 216.2 | 0.58 | 50 | 5.04 | 15 | 27.78 | 19.28 | 11.13 | 156.1 | 2.39 | 2.81 |
| **Cock-eyed squid, *Histioteuthis heteropsis*** | 1290.8 | 3.49 | 33 | 3.32 | 13 | 24.07 | 17.83 | 10.3 | 163.99 | 2.51 | 3.41 |
| ***Gonatus* spp.** | 24.1 | 0.06 | 29 | 2.92 | 15 | 27.78 | 17.76 | 10.25 | 82.93 | 1.27 | 1.49 |
| **Jack mackerel, *Trachurus symmetricus*** | 1461.4 | 3.95 | 43 | 4.33 | 11 | 20.37 | 16.54 | 9.55 | 168.66 | 2.58 | 4.14 |
| **Pacific sardine, *Sardinops sagax*** | 372.7 | 1.01 | 26 | 2.62 | 13 | 24.07 | 15.99 | 9.23 | 87.28 | 1.34 | 1.82 |
| ***Onychoteuthis borealijaponica*** | 502.6 | 1.36 | 18 | 1.81 | 13 | 24.07 | 15.73 | 9.08 | 76.34 | 1.17 | 1.59 |
| **Slender barracudina, *Lestidiops ringens*** | 26.8 | 0.07 | 28 | 2.82 | 12 | 22.22 | 14.5 | 8.37 | 64.27 | 0.98 | 1.45 |
| **Jumbo squid, *Dosidicus gigas*** | 1394.3 | 3.77 | 27 | 2.72 | 10 | 18.52 | 14.44 | 8.34 | 120.13 | 1.84 | 3.25 |
| **Pacific mackerel, *Scomber japonicus*** | 1583.7 | 4.28 | 54 | 5.44 | 8 | 14.81 | 14.16 | 8.18 | 143.97 | 2.2 | 4.86 |
| **Flowervase jewell squid, *Histioteuthis dofleini*** | 541.2 | 1.46 | 19 | 1.91 | 10 | 18.52 | 12.64 | 7.3 | 62.52 | 0.96 | 1.69 |
| **Sunbeam lampfish, *Lampadena urophaos*** | 127.9 | 0.35 | 22 | 2.22 | 8 | 14.81 | 10.03 | 5.79 | 37.94 | 0.58 | 1.29 |
| **Pacific pomfret, *Brama japonica*** | 622 | 1.68 | 8 | 0.81 | 6 | 11.11 | 7.85 | 4.53 | 27.63 | 0.42 | 1.25 |
| **Chubby pearleye, *Rosenblattichthys volucris*** | 26.5 | 0.07 | 14 | 1.41 | 5 | 9.26 | 6.2 | 3.58 | 13.72 | 0.21 | 0.74 |
| **Unidentified Scopelarchidae** | 19.8 | 0.05 | 29 | 2.92 | 4 | 7.41 | 5.99 | 3.46 | 22.03 | 0.34 | 1.49 |
| **Pacific saury, *Cololabis saira*** | 142.4 | 0.38 | 8 | 0.81 | 4 | 7.41 | 4.96 | 2.87 | 8.82 | 0.13 | 0.60 |
| **Unidentified Eucarida** | 1 | <0.01 | 25 | 2.52 | 1 | 1.85 | 2.52 | 1.46 | 4.67 | 0.07 | 1.27 |
| **California smoothtongue, *Leuroglossus stilbius*** | <0.1 | <0.01 | 3 | 0.3 | 2 | 3.7 | 2.31 | 1.34 | 1.12 | 0.02 | 0.16 |
| ***Argonauta* sp.** | 5.5 | 0.01 | 2 | 0.2 | 2 | 3.7 | 2.26 | 1.31 | 0.8 | 0.01 | 0.11 |
| ***Octopoteuthis* sp.** | 2 | 0.01 | 2 | 0.2 | 2 | 3.7 | 2.26 | 1.3 | 0.77 | 0.01 | 0.11 |
| **Luvar, *Luvarus imperialis*** | 471.9 | 1.28 | 1 | 0.1 | 1 | 1.85 | 1.86 | 1.08 | 2.55 | 0.04 | 0.69 |
| **Dogtooth lampfish, *Ceratoscopelus townsendi*** | 1.5 | <0.01 | 2 | 0.2 | 1 | 1.85 | 1.19 | 0.69 | 0.38 | 0.01 | 0.11 |
| **Unidentified Teuthoidea** | 7.5 | 0.02 | 1 | 0.1 | 1 | 1.85 | 1.14 | 0.66 | 0.22 | <0.01 | 0.06 |
| **Unidentified Tunicata** | 0.3 | <0.01 | 1 | 0.1 | 1 | 1.85 | 1.13 | 0.65 | 0.19 | <0.01 | 0.06 |
| ***Histioteuthis* spp.** | <0.1 | <0.01 | 1 | 0.1 | 1 | 1.85 | 1.13 | 0.65 | 0.19 | <0.01 | 0.06 |
| ***Chiroteuthis calyx*** | <0.1 | <0.01 | 1 | 0.1 | 1 | 1.85 | 1.13 | 0.65 | 0.19 | <0.01 | 0.06 |
| **Bigfin lampfish, *Symbolophorus californiensis*** | <0.1 | <0.01 | 1 | 0.1 | 1 | 1.85 | 1.13 | 0.65 | 0.19 | <0.01 | 0.06 |
| **California headlightfish, *Diaphus theta*** | <0.1 | <0.01 | 1 | 0.1 | 1 | 1.85 | 1.13 | 0.65 | 0.19 | <0.01 | 0.06 |
