# Supplementary material for: Feeding ecology of broadbill swordfish (Xiphias gladius) in the California current
Source: PLoS One. 2023 Feb 16;18(2):e0258011. doi: 10.1371/journal.pone.0258011 (PMC9934375; doi:10.1371/journal.pone.0258011)
Supplement: S12 Table — A total of 36 stomachs containing food was examined. Prey items are shown by decreasing GII value. See methods for description of the measured values. (DOCX) [file pone.0258011.s015.docx]

**Table S12.** Quantitative prey composition of the broadbill swordfish during year 2012 in the California Current. A total of 36 stomachs containing food was examined. Prey items are shown by decreasing GII value. See methods for description of the measured values.

| **Prey Species** | ***W* (g)** | ***%W*** | ***N*** | ***%N*** | ***F*** | ***%F*** | **GII** | **%GII** | **IRI** | **%IRI** | **%PSIRI** |
| --- | --- | --- | --- | --- | --- | --- | --- | --- | --- | --- | --- |
| **Jumbo squid, *Dosidicus gigas*** | 25904.7 | 61.32 | 229 | 37.79 | 27 | 75 | 100.52 | 58.04 | 7433.13 | 70.52 | 49.56 |
| **Boreopacific gonate squid, *Gonatopsis borealis*** | 2170.6 | 5.14 | 61 | 10.07 | 23 | 63.89 | 45.66 | 26.36 | 971.37 | 9.22 | 7.61 |
| ***Abraliopsis* sp.** | 38.9 | 0.09 | 69 | 11.39 | 20 | 55.56 | 38.7 | 22.34 | 637.67 | 6.05 | 5.74 |
| **Pacific hake, *Merluccius productus*** | 6452.3 | 15.27 | 41 | 6.77 | 10 | 27.78 | 28.76 | 16.61 | 612.2 | 5.81 | 11.02 |
| **Unidentified Teleostei** | 173.2 | 0.41 | 22 | 3.63 | 14 | 38.89 | 24.79 | 14.31 | 157.12 | 1.49 | 2.02 |
| **Market squid, *Doryteuthis opalescens*** | 30.6 | 0.07 | 49 | 8.09 | 10 | 27.78 | 20.75 | 11.98 | 226.62 | 2.15 | 4.08 |
| ***Gonatus* spp.** | 5.4 | 0.01 | 16 | 2.64 | 11 | 30.56 | 19.17 | 11.07 | 81.06 | 0.77 | 1.33 |
| **Jack mackerel, *Trachurus symmetricus*** | 4293 | 10.16 | 16 | 2.64 | 5 | 13.89 | 15.41 | 8.9 | 177.81 | 1.69 | 6.40 |
| **Chubby pearleye, *Rosenblattichthys volucris*** | 84 | 0.2 | 25 | 4.13 | 7 | 19.44 | 13.72 | 7.92 | 84.08 | 0.8 | 2.17 |
| **Duckbill barracudina, *Magnisudis atlantica*** | 265.6 | 0.63 | 16 | 2.64 | 7 | 19.44 | 13.11 | 7.57 | 63.56 | 0.6 | 1.64 |
| ***Onychoteuthis borealijaponica*** | 13.3 | 0.03 | 9 | 1.49 | 7 | 19.44 | 12.1 | 6.99 | 29.49 | 0.28 | 0.76 |
| **Pacific sardine, *Sardinops sagax*** | 414.5 | 0.98 | 8 | 1.32 | 4 | 11.11 | 7.74 | 4.47 | 25.57 | 0.24 | 1.15 |
| **Pacific saury, *Cololabis saira*** | 27.7 | 0.07 | 5 | 0.83 | 4 | 11.11 | 6.93 | 4 | 9.9 | 0.09 | 0.45 |
| **Slender barracudina, *Lestidiops ringens*** | 48.2 | 0.11 | 7 | 1.16 | 3 | 8.33 | 5.54 | 3.2 | 10.58 | 0.1 | 0.64 |
| **Cock-eyed squid, *Histioteuthis heteropsis*** | 21.3 | 0.05 | 7 | 1.16 | 3 | 8.33 | 5.51 | 3.18 | 10.05 | 0.1 | 0.61 |
| **Pacific mackerel, *Scomber japonicus*** | 451.6 | 1.07 | 2 | 0.33 | 2 | 5.56 | 4.02 | 2.32 | 7.77 | 0.07 | 0.70 |
| ***Argonauta* sp.** | 7.4 | 0.02 | 2 | 0.33 | 2 | 5.56 | 3.41 | 1.97 | 1.93 | 0.02 | 0.18 |
| ***Octopoteuthis* sp.** | <0.1 | <0.01 | 2 | 0.33 | 2 | 5.56 | 3.4 | 1.96 | 1.83 | 0.02 | 0.17 |
| **Splitnose rockfish, *Sebastes diploproa*** | 924.2 | 2.19 | 2 | 0.33 | 1 | 2.78 | 3.06 | 1.77 | 6.99 | 0.07 | 1.26 |
| ***Nansenia* spp.** | 4.7 | 0.01 | 10 | 1.65 | 1 | 2.78 | 2.56 | 1.48 | 4.61 | 0.04 | 0.83 |
| **Albacore, *Thunnus alalunga*** | 371.6 | 0.88 | 1 | 0.17 | 1 | 2.78 | 2.21 | 1.27 | 2.9 | 0.03 | 0.53 |
| **King-of-the-salmon, *Trachipterus altivelis*** | 331.4 | 0.78 | 1 | 0.17 | 1 | 2.78 | 2.15 | 1.24 | 2.64 | 0.03 | 0.48 |
| **Unidentified Teuthoidea** | 130.2 | 0.31 | 1 | 0.17 | 1 | 2.78 | 1.88 | 1.08 | 1.31 | 0.01 | 0.24 |
| **Halfmoon, *Medialuna californiensis*** | 81 | 0.19 | 1 | 0.17 | 1 | 2.78 | 1.81 | 1.04 | 0.99 | 0.01 | 0.18 |
| ***Cranchia scabra*** | <0.1 | <0.01 | 1 | 0.17 | 1 | 2.78 | 1.7 | 0.98 | 0.46 | <0.01 | 0.09 |
| ***Chiroteuthis calyx*** | <0.1 | <0.01 | 1 | 0.17 | 1 | 2.78 | 1.7 | 0.98 | 0.46 | <0.01 | 0.09 |
| **Mexican lampfish, *Triphoturus mexicanus*** | <0.1 | <0.01 | 1 | 0.17 | 1 | 2.78 | 1.7 | 0.98 | 0.46 | <0.01 | 0.09 |
| **California smoothtongue, *Leuroglossus stilbius*** | <0.1 | <0.01 | 1 | 0.17 | 1 | 2.78 | 1.7 | 0.98 | 0.46 | <0.01 | 0.09 |
