# Supplementary material for: Feeding ecology of broadbill swordfish (Xiphias gladius) in the California current
Source: PLoS One. 2023 Feb 16;18(2):e0258011. doi: 10.1371/journal.pone.0258011 (PMC9934375; doi:10.1371/journal.pone.0258011)
Supplement: S13 Table — A total of 56 stomachs containing food was examined. Prey items are shown by decreasing GII value. See methods for description of the measured values. (DOCX) [file pone.0258011.s016.docx]

**Table S13.** Quantitative prey composition of the broadbill swordfish during year 2013 in the California Current. A total of 56 stomachs containing food was examined. Prey items are shown by decreasing GII value. See methods for description of the measured values.

| **Prey Species** | ***W* (g)** | ***%W*** | ***N*** | ***%N*** | ***F*** | ***%F*** | **GII** | **%GII** | **IRI** | **%IRI** | **%PSIRI** |
| --- | --- | --- | --- | --- | --- | --- | --- | --- | --- | --- | --- |
| **Jumbo squid, *Dosidicus gigas*** | 27077.4 | 46.33 | 273 | 26.25 | 48 | 85.71 | 91.39 | 52.77 | 6221.25 | 68.78 | 36.29 |
| **Boreopacific gonate squid, *Gonatopsis borealis*** | 3122.2 | 5.34 | 97 | 9.33 | 30 | 53.57 | 39.4 | 22.75 | 785.85 | 8.69 | 7.34 |
| ***Abraliopsis* sp.** | 4.1 | 0.01 | 108 | 10.38 | 28 | 50 | 34.87 | 20.13 | 519.58 | 5.74 | 5.20 |
| ***Gonatus* spp.** | 36.9 | 0.06 | 56 | 5.38 | 24 | 42.86 | 27.89 | 16.1 | 233.48 | 2.58 | 2.72 |
| **Market squid, *Doryteuthis opalescens*** | 484.7 | 0.83 | 146 | 14.04 | 16 | 28.57 | 25.08 | 14.48 | 424.79 | 4.7 | 7.44 |
| **Luvar, *Luvarus imperialis*** | 18590.4 | 31.81 | 16 | 1.54 | 5 | 8.93 | 24.41 | 14.09 | 297.75 | 3.29 | 16.68 |
| ***Onychoteuthis borealijaponica*** | 136.7 | 0.23 | 31 | 2.98 | 13 | 23.21 | 15.26 | 8.81 | 74.63 | 0.82 | 1.61 |
| **Pacific hake, *Merluccius productus*** | 2686.3 | 4.6 | 72 | 6.92 | 8 | 14.29 | 14.9 | 8.6 | 164.56 | 1.82 | 5.76 |
| **Unidentified Teleostei** | 61.4 | 0.11 | 17 | 1.63 | 14 | 25 | 15.44 | 8.91 | 8.91 | 0.1 | 0.87 |
| **Chubby pearleye, *Rosenblattichthys volucris*** | 348.5 | 0.6 | 35 | 3.37 | 10 | 17.86 | 12.6 | 7.27 | 70.74 | 0.78 | 1.99 |
| **Duckbill barracudina, *Magnisudis atlantica*** | 746.4 | 1.28 | 18 | 1.73 | 10 | 17.86 | 12.05 | 6.96 | 53.71 | 0.59 | 1.51 |
| **King-of-the-salmon, *Trachipterus altivelis*** | 1854.6 | 3.17 | 13 | 1.25 | 7 | 12.5 | 9.77 | 5.64 | 55.29 | 0.61 | 2.21 |
| **Pacific pomfret, *Brama japonica*** | 1471.4 | 2.52 | 13 | 1.25 | 7 | 12.5 | 9.39 | 5.42 | 47.1 | 0.52 | 1.89 |
| **Pacific saury, *Cololabis saira*** | 1069 | 1.83 | 47 | 4.52 | 3 | 5.36 | 6.76 | 3.9 | 34.01 | 0.38 | 3.18 |
| ***Histioteuthis* spp.** | 56.5 | 0.1 | 6 | 0.58 | 6 | 10.71 | 6.57 | 3.8 | 7.22 | 0.08 | 0.34 |
| **Slender barracudina, *Lestidiops ringens*** | 8.9 | 0.02 | 10 | 0.96 | 5 | 8.93 | 5.72 | 3.3 | 8.72 | 0.1 | 0.49 |
| **Pacific sardine, *Sardinops sagax*** | 392.9 | 0.67 | 13 | 1.25 | 4 | 7.14 | 5.23 | 3.02 | 13.73 | 0.15 | 0.96 |
| **Sunbeam lampfish, *Lampadena urophaos*** | 59.2 | 0.1 | 9 | 0.87 | 3 | 5.36 | 3.65 | 2.11 | 5.18 | 0.06 | 0.49 |
| **Unidentified Euphausiidae** | 3 | 0.01 | 6 | 0.58 | 3 | 5.36 | 3.43 | 1.98 | 3.12 | 0.03 | 0.30 |
| **Robust clubhook squid*, Onykia robusta*** | 43.3 | 0.07 | 4 | 0.38 | 3 | 5.36 | 3.36 | 1.94 | 2.46 | 0.03 | 0.23 |
| **Unidentified Scopelarchidae** | 96.3 | 0.16 | 18 | 1.73 | 2 | 3.57 | 3.16 | 1.82 | 6.77 | 0.07 | 0.95 |
| ***Nansenia* spp.** | 9.4 | 0.02 | 3 | 0.29 | 2 | 3.57 | 2.24 | 1.29 | 1.09 | 0.01 | 0.16 |
| **Flowervase jewell squid, *Histioteuthis dofleini*** | 16.1 | 0.03 | 2 | 0.19 | 2 | 3.57 | 2.19 | 1.26 | 0.79 | 0.01 | 0.11 |
| ***Sebastes* spp.** | 3 | 0.01 | 8 | 0.77 | 1 | 1.79 | 1.48 | 0.85 | 1.38 | 0.02 | 0.39 |
| **Unidentified Eucarida** | 0.1 | <0.01 | 6 | 0.58 | 1 | 1.79 | 1.36 | 0.79 | 1.03 | 0.01 | 0.30 |
| **Unidentified Teuthoidea** | <0.1 | <0.01 | 2 | 0.19 | 1 | 1.79 | 1.14 | 0.66 | 0.34 | <0.01 | 0.10 |
| ***Leachia dislocata*** | <0.1 | <0.01 | 2 | 0.19 | 1 | 1.79 | 1.14 | 0.66 | 0.34 | <0.01 | 0.10 |
| **Pacific mackerel, *Scomber japonicus*** | 46.4 | 0.08 | 1 | 0.1 | 1 | 1.79 | 1.13 | 0.65 | 0.31 | <0.01 | 0.09 |
| **Jack mackerel, *Trachurus symmetricus*** | 15.7 | 0.03 | 1 | 0.1 | 1 | 1.79 | 1.1 | 0.64 | 0.22 | <0.01 | 0.07 |
| **Unidentified Tunicata** | 2 | <0.01 | 1 | 0.1 | 1 | 1.79 | 1.09 | 0.63 | 0.18 | <0.01 | 0.06 |
| ***Argonauta* sp.** | 0.1 | <0.01 | 1 | 0.1 | 1 | 1.79 | 1.09 | 0.63 | 0.17 | <0.01 | 0.06 |
| ***Octopoteuthis* sp.** | <0.1 | <0.01 | 1 | 0.1 | 1 | 1.79 | 1.09 | 0.63 | 0.17 | <0.01 | 0.06 |
| ***Octopus* spp.** | <0.1 | <0.01 | 1 | 0.1 | 1 | 1.79 | 1.09 | 0.63 | 0.17 | <0.01 | 0.06 |
| **Bigfin lampfish, *Symbolophorus californiensis*** | <0.1 | <0.01 | 1 | 0.1 | 1 | 1.79 | 1.09 | 0.63 | 0.17 | <0.01 | 0.06 |
| **Spotted barracudina, *Arctozenus risso*** | <0.1 | <0.01 | 1 | 0.1 | 1 | 1.79 | 1.09 | 0.63 | 0.17 | <0.01 | 0.06 |
| **Unidentified Isopoda** | <0.1 | <0.01 | 1 | 0.1 | 1 | 1.79 | 1.09 | 0.63 | 0.17 | <0.01 | 0.06 |
