# Supplementary material for: Feeding ecology of broadbill swordfish (Xiphias gladius) in the California current
Source: PLoS One. 2023 Feb 16;18(2):e0258011. doi: 10.1371/journal.pone.0258011 (PMC9934375; doi:10.1371/journal.pone.0258011)
Supplement: S14 Table — A total of 34 stomachs containing food was examined. Prey items are shown by decreasing GII value. See methods for description of the measured values. (DOCX) [file pone.0258011.s017.docx]

**Table S14.** Quantitative prey composition of the broadbill swordfish during year 2014 in the California Current. A total of 34 stomachs containing food was examined. Prey items are shown by decreasing GII value. See methods for description of the measured values.

| **Prey Species** | ***W* (g)** | ***%W*** | ***N*** | ***%N*** | ***F*** | ***%F*** | **GII** | **%GII** | **IRI** | **%IRI** | **%PSIRI** |
| --- | --- | --- | --- | --- | --- | --- | --- | --- | --- | --- | --- |
| **Pacific hake, *Merluccius productus*** | 5071.2 | 47.06 | 44 | 8.12 | 10 | 29.41 | 48.84 | 28.2 | 51152.2 | 51.71 | 27.59 |
| **Boreopacific gonate squid, *Gonatopsis borealis*** | 490 | 4.55 | 43 | 7.93 | 18 | 52.94 | 37.77 | 21.81 | 9593.82 | 9.7 | 6.24 |
| **Duckbill barracudina, *Magnisudis atlantica*** | 581.1 | 5.39 | 38 | 7.01 | 15 | 44.12 | 32.63 | 18.84 | 9286.95 | 9.39 | 6.20 |
| **Chubby pearleye, *Rosenblattichthys volucris*** | 228.6 | 2.12 | 61 | 11.25 | 12 | 35.29 | 28.1 | 16.22 | 3475.2 | 3.51 | 6.69 |
| **King-of-the-salmon, *Trachipterus altivelis*** | 3391.4 | 31.47 | 11 | 2.03 | 5 | 14.71 | 27.83 | 16.07 | 17012 | 17.2 | 16.75 |
| ***Gonatus* spp.** | 82.2 | 0.76 | 39 | 7.2 | 13 | 38.24 | 26.67 | 15.4 | 1575.08 | 1.59 | 3.98 |
| **Unidentified Eucarida** | 4.4 | 0.04 | 123 | 22.69 | 4 | 11.76 | 19.92 | 11.5 | 509.6 | 0.52 | 11.37 |
| **Slender barracudina, *Lestidiops ringens*** | 246.1 | 2.28 | 45 | 8.3 | 8 | 23.53 | 19.7 | 11.37 | 2328.8 | 2.35 | 5.29 |
| ***Nansenia* spp.** | 221.9 | 2.06 | 29 | 5.35 | 9 | 26.47 | 19.56 | 11.29 | 2258.28 | 2.28 | 3.71 |
| **Market squid, *Doryteuthis opalescens*** | 8.6 | 0.08 | 33 | 6.09 | 9 | 26.47 | 18.84 | 10.88 | 374.04 | 0.38 | 3.09 |
| **Spotted barracudina, *Arctozenus risso*** | 67.9 | 0.63 | 13 | 2.4 | 7 | 20.59 | 13.64 | 7.87 | 566.37 | 0.57 | 1.52 |
| **Unidentified Teleostei** | 48.7 | 0.45 | 10 | 1.85 | 6 | 17.65 | 11.51 | 6.65 | 40.53 | 0.04 | 1.15 |
| **Sunbeam lampfish, *Lampadena urophaos*** | 7.5 | 0.07 | 8 | 1.48 | 4 | 11.76 | 7.68 | 4.44 | 62.08 | 0.06 | 0.78 |
| **Pacific saury, *Cololabis saira*** | 36.2 | 0.34 | 6 | 1.11 | 4 | 11.76 | 7.63 | 4.4 | 168.8 | 0.17 | 0.73 |
| **Unidentified Teuthoidea** | 56 | 0.52 | 4 | 0.74 | 3 | 8.82 | 5.82 | 3.36 | 180.03 | 0.18 | 0.63 |
| ***Cranchia scabra*** | 4.5 | 0.04 | 4 | 0.74 | 3 | 8.82 | 5.54 | 3.2 | 25.56 | 0.03 | 0.39 |
| ***Argonauta* sp.** | 0.1 | <0.01 | 3 | 0.55 | 3 | 8.82 | 5.41 | 3.13 | 9.36 | 0.01 | 0.28 |
| ***Abraliopsis* sp.** | <0.1 | <0.01 | 3 | 0.55 | 3 | 8.82 | 5.41 | 3.13 | 9.09 | 0.01 | 0.28 |
| **Mexican lampfish, *Triphoturus mexicanus*** | <0.1 | <0.01 | 3 | 0.55 | 3 | 8.82 | 5.41 | 3.13 | 9.09 | 0.01 | 0.28 |
| **Bigfin lampfish, *Symbolophorus californiensis*** | 1.7 | 0.02 | 4 | 0.74 | 2 | 5.88 | 3.83 | 2.21 | 11.42 | 0.01 | 0.38 |
| **Jack mackerel, *Trachurus symmetricus*** | 7.6 | 0.07 | 2 | 0.37 | 2 | 5.88 | 3.65 | 2.11 | 19.2 | 0.02 | 0.22 |
| ***Onychoteuthis borealijaponica*** | 4 | 0.04 | 2 | 0.37 | 2 | 5.88 | 3.63 | 2.1 | 12 | 0.01 | 0.21 |
| **Jumbo squid, *Dosidicus gigas*** | <0.1 | <0.01 | 2 | 0.37 | 2 | 5.88 | 3.61 | 2.08 | 4.04 | <0.01 | 0.19 |
| **Luvar, *Luvarus imperialis*** | 196.2 | 1.82 | 1 | 0.18 | 1 | 2.94 | 2.86 | 1.65 | 197.2 | 0.2 | 1.00 |
| ***Japetella* sp.** | <0.1 | <0.01 | 3 | 0.55 | 1 | 2.94 | 2.02 | 1.16 | 3.01 | <0.01 | 0.28 |
| **Smalleye squaretail, *Tetragonurus cuvieri*** | 13.7 | 0.13 | 2 | 0.37 | 1 | 2.94 | 1.98 | 1.15 | 15.7 | 0.02 | 0.25 |
| **Unidentified Scopelarchidae** | 6.2 | 0.06 | 2 | 0.37 | 1 | 2.94 | 1.94 | 1.12 | 8.2 | 0.01 | 0.22 |
| **Unidentified Tunicata** | 1.2 | 0.01 | 1 | 0.18 | 1 | 2.94 | 1.81 | 1.05 | 2.2 | <0.01 | 0.10 |
| **Pacific mackerel, *Scomber japonicus*** | <0.1 | <0.01 | 1 | 0.18 | 1 | 2.94 | 1.8 | 1.04 | 1.01 | <0.01 | 0.10 |
| ***Octopus rubescens*** | <0.1 | <0.01 | 1 | 0.18 | 1 | 2.94 | 1.8 | 1.04 | 1.01 | <0.01 | 0.10 |
| **Cock-eyed squid, *Histioteuthis heteropsis*** | <0.1 | <0.01 | 1 | 0.18 | 1 | 2.94 | 1.8 | 1.04 | 1.01 | <0.01 | 0.10 |
