# Supplementary material for: Feeding ecology of broadbill swordfish (Xiphias gladius) in the California current
Source: PLoS One. 2023 Feb 16;18(2):e0258011. doi: 10.1371/journal.pone.0258011 (PMC9934375; doi:10.1371/journal.pone.0258011)
Supplement: S15 Table — Values of mean GII, bootstrapped 95% CIs and % bootstrap runs in which each prey type was in each of two categories of swordfish. If more than 95% (or fewer than 5%) of runs show the prey type was more important in one year than the other, the difference is considered to be significant. Y1 = Year1 (2007), Y2 = Year2 (2008–2010), Y3 = Year3 (2011–2014). These results are generally consistent with inferences from non-overlap of 95% CIs. (DOCX) [file pone.0258011.s018.docx]

**Table S15.** Comparison of GII for the main prey species for broadbill swordfish by year group. Values of mean GII, bootstrapped 95% CIs and % bootstrap runs in which each prey type was in each of two categories of swordfish. If more than 95% (or fewer than 5%) of runs show the prey type was more important in one year than the other, we consider the difference to be significant. Y1 = Year1 (2007), Y2 = Year2 (2008-2010), Y3 = Year3 (2011-2014). These results are generally consistent with inferences from non-overlap of 95% CIs.

|  | **Mean GII and (95% CI)** | |  | **Bootstrap results** | | |
| --- | --- | --- | --- | --- | --- | --- |
| **Prey taxa** | **Y1** | **Y2** | **Y3** | **% runs**  **Y1>Y2** | **% runs**  **Y1>Y3** | **% runs**  **Y2>Y3** |
| **Jumbo squid** | 115.36  (100.85 - 128.86) | 98.02  (84.40 - 109.08) | 58.50  (48.66 - 67.84) | 97.3 | 100.0 | 100.0 |
| ***Gonatopsis borealis*** | 52.34  (39.54 - 67.01) | 73.95  (64.31 - 83.94) | 40.62  (34.98 - 45.76) | 0.8 | 94.4 | 100.0 |
| ***Abraliopsis* sp.** | 23.92  (12.98 - 35.13) | 32.26  (23.55 - 40.68) | 27.96  (23.33 - 33.13) | 10.9 | 23.0 | 80.4 |
| ***Gonatus* spp.** | 22.27  (13.04 - 30.70) | 32.53  (24.54 - 40.80) | 23.38  (18.96 - 27.86) | 4.7 | 42.6 | 97.6 |
| **Market squid** | 5.64  (1.30 - 10.79) | 28.93  (19.55 - 37.96) | 26.05  (20.59 - 32.04) | 0.0 | 0.0 | 67.3 |
| **Pacific hake** | 1.40  (0.00 - 4.47) | 0.00  (0.00 - 0.00) | 35.68  (25.50 - 44.92) | 63.9 | 0.0 | 0.0 |
