# Supplementary material for: Feeding ecology of broadbill swordfish (Xiphias gladius) in the California current
Source: PLoS One. 2023 Feb 16;18(2):e0258011. doi: 10.1371/journal.pone.0258011 (PMC9934375; doi:10.1371/journal.pone.0258011)
Supplement: Attachment — Submitted filename: Response to Reviewers.docx [file pone.0258011.s020.docx]

Dr. Antonio Medina Guerrero
Academic Editor
PLOS ONE

Dear Dr. Medina Guerrero,

Thank you for the opportunity to submit a second revised draft of our manuscript titled: “Feeding ecology of broadbill swordfish (*Xiphias gladius*) in the California Current”**.** Once again we appreciate the time and effort that you and the reviewers have dedicated to providing your valuable feedback and insightful comments to our manuscript. We have incorporated the new changes to reflect the suggestions reviewers provided in the best way we could. We have highlighted our proposed changes to the manuscript through MS Word’s ‘track changes’ feature. Here is a point-by-point response to the reviewers’ new comments and concerns.

Thank you so much for your help and your time.

Sincerely,

Antonella Preti and coauthors

Minor comments
Introduction:

Line 49: What do you mean with “they are productive”? Perhaps you wanted to say that they are highly abundant or that they are meso-predators?

**We decided to remove the statement as it is not central to the topic.**

Line 99-119: I appreciate the explanation that authors have included to answer my comment of being more concrete on how their findings can be used. The text is highly complete, but very long. Perhaps you could summarize the text with something similar to:

Due to the complexity of many ecosystems, there is a need for basic knowledge of trophic interactions that are critical to understand system productivity and food chain dynamics. New policy developments have increased the relevance of feeding ecology studies, as policy-makers and fisheries managers have embraced the concept of ecosystem-based fisheries management (EBFM), thus taking a more holistic approach to resource management [34, 35]. The findings of this study can serve to inform ecosystem models considering trophic interactions and contribute to the development of alternative approaches to better manage this economically and ecologically important species and move towards an EBFM.

**We thank the reviewer for the detailed explanation and help. In line with the suggestion, we updated this section as follows:**

**“Due to the complexity of many ecosystems, there is a need for basic knowledge of trophic interactions that are critical to understand system productivity and food chain dynamics. New policy developments have increased the relevance of feeding ecology studies, as policy-makers and fisheries managers have embraced the concept of ecosystem-based fisheries management (EBFM), thus taking a more holistic approach to resource management [34, 35]. The findings of this study can inform ecosystem models with information about trophic interactions, contributing to the development of alternative approaches to better manage this economically and ecologically important species.”**


Material and Methods:
The lack of ecological criteria to divide large and small individuals and to divide the half year in two equal periods, in my opinion is not the best option, but considering that how the analysis was conducted is well explained and the authors have justified the use of this criteria, I will not insist in that aspect.

**We thank the reviewer for sharing their perspective.**

Lines 291-294: If DE is not used for model selection, it is not necessary to give all of this explanation on the use of DE, and these sentences can be removed.

**We removed the sentences as suggested, and slightly modified the subsequent sentence to read, “The AIC trades off higher values of the likelihood function against a penalty for adding more parameters.”**


Lines 230-231: Since in the discussion you relate differences in diet with the characteristics of the sampling area (within or beyond SCB), please give some information/description of the difference between the SCB area and the rest of the sampling area. For example, include if SCB is a shallower area or influenced with specific currents, and if SCB is considered more inshore than the rest as mentioned in the discussion (lines 565-568). For somebody not familiar with the area of study, SCB sampling points do not look more inshore than the rest of the sampling points.

**We revised the language to clarify the difference between the SCB area and currents compared to the rest of the sampling area, as follows: “…‘within the SCB’ (east of 120º 30’W longitude) and ‘beyond the SCB’ (west of 120º 30’W longitude), reflecting separation between the more inshore waters in the SCB where the northward flowing California Counter Current influences nearshore oceanography and the more offshore waters affected by the California Current as it moves southward…”**

Results:
Line 404: The description of the legend (Red= Teuthoidea; Blue = Teleostei…) is already described in the figure 4 caption, therefore it can be deleted from the body text.

**We deleted the text as suggested.**

Line 447: Thank you for including the R-adj sq values. Considering these values, I wanted to add an additional comment. In the GAM model for *Abraliopsis* sp., the R-sq (adj.) is negative indicating that the model is not good or better than a straight line. Then, this model should not be considered as valid.

**While we concede the reviewer’s point that the GAM model for *Abraliopsis* sp. did not explain much of the variation in swordfish diet, we nonetheless include it in our results for completeness. We interpret the negative R-sq (adj.) to indicate a very weak or nonexistent relationship between *Abraliopsis* sp. consumption and the explanatory variables in our model. We added a statement in the manuscript that the** ***Abraliopsis* sp. model was unsatisfactory as indicated by the negative R-square adjusted.**


Discussion:
Thank you for addressing my comments, but still I don’t see the connection of the paragraph 480-489 with your results, please can you connect this first paragraph with the discussion of your results?

**To make this connection, we added the following sentence at the beginning of the paragraph: “The range of prey species found in our study is consistent with the diurnal vertical distribution of swordfish, reflecting their diving behavior.”**

Reviewer #2: (No Response)

Reviewer #3: (No Response)

7. PLOS authors have the option to publish the peer review history of their article. If published, this will include your full peer review and any attached files.


If you choose “no”, your identity will remain anonymous but your review may still be made public.

**No. We would prefer not to have this option.**

Do you want your identity to be public for this peer review? For information about this choice, including consent withdrawal, please see our [Privacy Policy](https://www.plos.org/privacy-policy).

Reviewer #1: No

Reviewer #2: **Yes:** Hiroshi Ohizumi

Reviewer #3: No

**We have added mention of Hiroshi Ohizumi’s contribution as a reviewer to our acknowledgments.**

[NOTE: If reviewer comments were submitted as an attachment file, they will be attached to this email and accessible via the submission site. Please log into your account, locate the manuscript record, and check for the action link "View Attachments". If this link does not appear, there are no attachment files.]

While revising your submission, please upload your figure files to the Preflight Analysis and Conversion Engine (PACE) digital diagnostic tool, <https://pacev2.apexcovantage.com/>. PACE helps ensure that figures meet PLOS requirements. To use PACE, you must first register as a user. Registration is free. Then, login and navigate to the UPLOAD tab, where you will find detailed instructions on how to use the tool. If you encounter any issues or have any questions when using PACE, please email PLOS at [figures@plos.org](mailto:figures@plos.org). Please note that Supporting Information files do not need this step.
